# Metallic‐Ion Controlled Dynamic Bonds to Co‐Harvest Isomerization Energy and Bond Enthalpy for High‐Energy Output of Flexible Self‐Heated Textile

**DOI:** 10.1002/advs.202201657

**Published:** 2022-05-01

**Authors:** Hui Wang, Yiyu Feng, Jian Gao, Wenyu Fang, Jing Ge, Xiaoyu Yang, Fei Zhai, Yunfei Yu, Wei Feng

**Affiliations:** ^1^ School of Materials Science and Engineering Tianjin University Tianjin 300350 China; ^2^ Key Laboratory of Materials Processing and Mold Ministry of Education Zhengzhou University Zhengzhou Henan 450002 China

**Keywords:** bond enthalpy, energy‐storage, flexibility, photothermal

## Abstract

Molecular light‐harvesting capabilities and the production of low‐temperature heat output are essential for flexible self‐heated textiles. An effective strategy to achieve these characteristics is to introduce photoresponsive molecular interactions (photodynamic bonds) to increase the energy storage capacity and optimize the low‐temperature photochromic kinetics. In this study, a series of sulfonic‐grafted azobenzene‐based polymers interacted with different metal ions (PAzo‐M, M = Mg, Ca, Ni, Zn, Cu, and Fe) to optimize the energy level and isomerization kinetics of these polymers is designed and prepared. Photoinduced formation and dissociation of M—O dynamic bonds enlarge the energy gap (∆*E*) between *trans* and *cis* isomers for high‐energy storage and favor a high rate of isomerization for low‐temperature heat release. The suitable binding energy and high ∆*E* enable PAzo‐M to store and release isomerization energy and bond enthalpy even in a low‐temperature (−5 °C) environment. PAzo‐Mg possesses the highest energy storage density of 408.6 J g^−1^ (113.5 Wh kg^−1^). A flexible textile coated with PAzo‐Mg can provide a high rise in temperature of 7.7–12.5 °C in a low‐temperature (−5.0 to 5.0 °C) environment by selectively self‐releasing heat indoors and outdoors. The flexible textile provides a new pathway for wearable thermal management devices.

## Graphical abstract

A series of photoresponsive azobenzene‐based polymers interacted with different metal ions show excellent photothermal energy storage and low‐temperature output capabilities through co‐harvesting of isomerization energy and bond enthalpy. The flexible self‐heated textile coated with azobenzene‐based polymers with dynamic bond can be fabricated for thermal management wearable device to realize temperature control in low‐temperature environments.

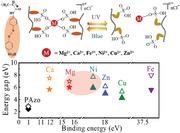

## 1 Introduction

Flexible self‐heated textiles with smart temperature controllability are basic components of smart flexible devices,^[^ ^1^ ^]^ on‐skin clothing,^[^ ^2^ ^]^ and soft robotics.^[^ ^3^ ^]^ Energy harvesting and utilization are essential for the continuous and controllable heat output of flexible self‐heated textiles, particularly in low‐temperature environments.^[^ ^4^, ^5^ ^]^ Smart self‐heated textiles have a unique potential advantage of cyclically using light energy through light‐harvesting, low‐temperature storage, and controllable heat release instead of through the photothermal effect in electric‐heating conductors and heat insulators.^[^ ^6^, ^7^, ^8^ ^]^ Polymer‐templated azobenzene is an ideal candidate for a photoresponsive material that can uniformly coat flexible self‐heated textiles owing to the combination of unique reversible *trans* (E) to *cis* (Z) photoisomerization and optimizable molecular interface interactions between the polymer and textile.^[^ ^4^, ^5^ ^]^ However, limited molecular levels, low energy capacity (<200.0 J g^−1^),^[^ ^9^, ^10^ ^]^ and the poor ability to release heat at low temperatures (a low rate of isomerization)^[^ ^11^, ^12^ ^]^ typically restrict the application of polymer‐templated azobenzene in self‐heated textiles. Therefore, there is presently a lack of flexible self‐heated textiles that can cyclically use photothermal energy for thermal management at low temperatures.

Introducing supramolecular interaction into azobenzene chromophore is an effective strategy to tune their molecular energy levels.^[^ ^13^, ^14^, ^15^, ^16^, ^17^ ^]^ Reversible isomerization‐induced formation and dissociation of supramolecular bonds enable polymer‐templated azobenzene to store and release additional bond enthalpy. Moreover, a lower thermal barrier for the Z‐to‐E reversion of metastable isomers with dangling bonds (dissociation) is achieved. The binding energy of new supramolecular bonds and isomerization degree are two vital parameters for achieving high energy storage capacity and rapid heat release at low temperatures.^[^ ^17^ ^]^ However, the trade‐off between the parameters typically reduces the energy utilization efficiency. Specifically, strong bonds enlarge the energy gap between the Z and E‐isomers by introducing bond enthalpy, but the cross‐linked chains might restrict the E‐to‐Z structural transformation (heat storage) due to the increased steric hindrance.^[^ ^18^ ^]^ Thus, supramolecular interactions with adjustable binding energies and the optimized structure of polymers are necessary for maximizing the utilization of isomerization energy and bond enthalpy of polymer‐templated azobenzene.

Dynamic bonds (dynamic ionic interactions and metal‐ligand interactions) are an important supramolecular interaction for optimizing molecular energy levels.^[^ ^19^, ^20^, ^21^ ^]^ The binding energy of dynamic bonds can be easily tuned by changing the metallic cations and organic groups. Li et al.^[^ ^22^ ^]^ summarized a wide range of metallic cations (arising from the s, p, d, or f blocks) and complexing groups (either neutral: amine, pyridine, nitrile, etc., or anionic: carboxylate, phosphonate, phenolate) according to the hard–soft acid–base (HSAB) theory and Lewis acid/base theory.^[^ ^23^ ^]^ The results indicated that these dynamic bonds are highly tunable. The dynamic‐bond‐controlled reversible crosslinking offers the potential for utilizing bond enthalpy in azobenzene‐based dynamic polymers. Additionally, the steric configuration and structural transformation of interacted azobenzene grafted on flexible chains are affected. Thus, this effect plays a vital role in temperature‐dependent isomerization kinetics for heat output. Wu^[^ ^24^ ^]^ reported photoresponsive metallopolymer organo hydrogels. The Ru–thioether dynamic bonds dissociated under light irradiation and reformed reversibly in the dark, thereby resulting in alternating crosslinking densities in the polymer networks. This process enabled the metallopolymer to undergo reversible gel‐to‐sol transitions even at −20 °C and self‐healing to control its mechanical properties. However, this study only reported the dissociation and re‐formation of the Ru–thioether dynamic bonds, and did not report on the change in energy levels and photothermal properties.

To the best of our knowledge, there is limited research on optimizing the isomerization energy and dynamic‐bond enthalpy of polymer‐templated azobenzene by controlling metal‐group interactions owing to the structural complexity in isomerization among reversible crosslinking polymers.^[^ ^25^ ^]^ Consequently, only a few photoresponsive polymer‐based textiles have realized self‐heating at a low temperature by cyclically using photothermal energy.^[^ ^18^ ^]^ Thus, the superior characteristics of flexible self‐heated textiles in smart temperature control applications have rarely been prepared or systematically investigated.

Herein, we present a strategy to control photodynamic bonds to molecularly optimize the energy levels of sulfonate‐azobenzene grafted on polyacrylic acid (PAzo) interacted with a variety of metallic (Mg^2+^, Ca^2+^, Ni^2+^, Zn^2+^, Cu^2+^, and Fe^3+^) ions. The dynamic bonds include dynamic ionic bonds and metal–ligand bonds, while alkali metal ions tend to form ionic bonds and transition metal ions tend to form metal–ligand bonds. Photocontrolled reversible formation and dissociation of dynamic bonds enlarge the energy gap between the E‐ and Z‐isomers for high‐energy storage. The effect of dynamic bonds with different binding energies on the degree and kinetics of photoisomerization and bond enthalpy is investigated in this study. The dynamic interactions also lower thermal barrier for Z‐to‐E isomerization and result in reversible dynamic bonds crosslinking of polymer chains. Cyclic utilization of isomerization energy and dynamic‐bond enthalpy enables PAzo‐M to show a high‐rate structural transformation at low temperature and release high‐power heat under light irradiation. Finally, we demonstrate the controllable self‐heated performance of PAzo‐M coating on a flexible textile in a low‐temperature (−5.0 to 5.0 °C) environment. The flexible self‐heated textile shows a great potential for smart flexible wearable devices.

## 2 Results and Discussion

### 2.1 Structural Design

**Figure 1:**
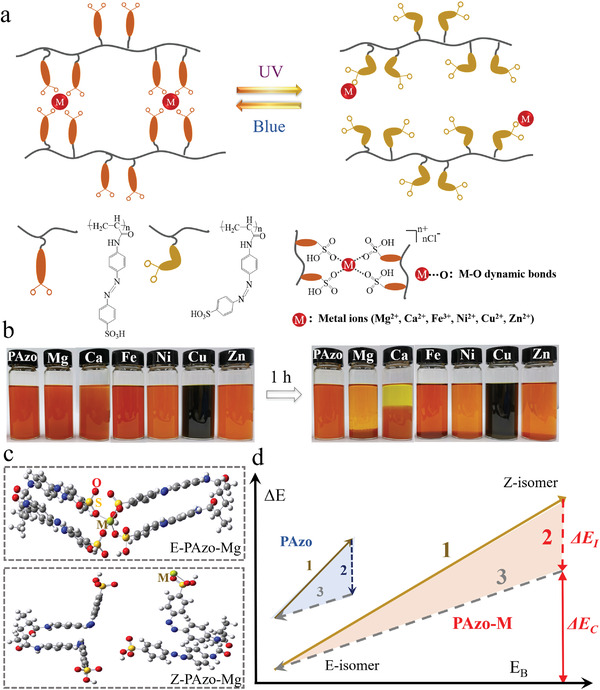
a) Chemical structure and schematic illustration of PAzo‐M with isomerization‐induced formation and dissociation of M—O dynamic bonds formed by different metallic ions, while *n* is 2 or 3. b) Photographs of PAzo solution after adding the same molar amount of MCl*_n_* (MgCl_2_, CaCl_2_, FeCl_3_, NiCl_2_, CuCl_2_, ZnCl_2_) for 1.0 h. c) Optimized geometric structure of PAzo‐Mg with the E‐ and Z‐isomers. d) Schematic energy diagram of PAzo and PAzo‐M during the energy utilization process of (1)–(3). Note: (1) E‐to‐Z isomerization, (2) Z‐to‐E isomerization, (3) bond enthalpy release by forming dynamic bonds or H‐bonds.

Intermolecular crosslinking between adjacent azobenzene chromophores in the polymer chains are important for controlling steric configuration, isomerization kinetics, and the molecular energy levels of both E‐ and Z‐isomers. Isomerization‐induced reversible formation and dissociation of dynamic bonds enable azobenzene molecules to co‐harvest isomerization energy and bond energy. The mechanism is shown in the **Figure** **1**a, PAzo‐M absorbs UV light (365 nm, 80 mW cm^−2^) to occur E‐to‐Z isomerization and dynamic bonds dissociate, while photoenergy is converted into latent chemical energy. Then, the isomerized PAzo‐M absorbs blue light (450 nm, 40 mW cm^−2^) to occur Z‐to‐E isomerization and dynamic bonds form with thermal relaxation.

To demonstrate the effect of dynamic bonds on the ability to harvest energy, we designed a series of azobenzene‐based polymers interacted with different metal ions. Optimizing dynamic bonds with different binding energies is an effective strategy to control intermolecular crosslinking, favor isomerization, and use bond enthalpy. Therefore, high‐energy low‐temperature heat release for self‐heating textiles can be realized. PAzo‐M (M = Mg, Ca, Ni, Zn, Cu, Fe) is prepared by crosslinking sulfonate‐azobenzene (Azo) grafted on PAzo using a variety of metal (M)─ group (—SO_3_H) dynamic bonds (Figure 1a). For the convenience of understanding, we define the “metal (M)─ group (—SO_3_H) dynamic bonds” as “M—O dynamic bonds”. The photographs of metal ions interacted with —SO_3_H group were shown in Figure 1b. The synthetic protocols and characterizations are presented in Figures S1–S10 (Supporting Information). The average molecular weight (*M*_n_) of PAzo is 2.2 × 10^4^ g mol^−1^, which indicates the presence of an average of ≈66 monomeric units (mAzo) per polymer chain (Figure S3, Supporting Information). ^1^H nuclear magnetic resonance (NMR), Fourier transform infrared (FTIR) spectra, and X‐ray photoelectron spectroscopy (XPS) spectra revealed that PAzo‐M were successfully prepared (details were presented in the Supporting Information). Unlike with previous hard templates,^[^ ^26^, ^27^ ^]^ a long flexible chain is potentially conducive to rapid isomerization at a relatively low temperature owing to the reduced steric hindrance.

The XPS spectra of PAzo‐M show that the atomic ratio of S:M is ≈4:1, which implies that the M—O dynamic bonds may have been formed by four —SO_3_H groups grafted on the polymer chains of E‐isomers with metallic ions. The increase in the number of M—O dynamic bonds is limited by the steric hindrance (Figures S7 and S8, Table S1, Supporting Information). The shift in the binding energy of O 1s and M of PAzo‐M controlled by photoisomerization indicates the reversible formation and dissociation of M—O dynamic bonds rather than M—N dynamic bonds with the unchanging of the binding energy of N 1s (Figures S9 and S10, Supporting Information). Typically, when the Mg—O dynamic bonds form among E‐PAzo‐Mg, the binding energy of O 1s is 532.3 eV. After the E‐to‐Z isomerization, the bonds dissociate in Z‐PAzo‐Mg, and the peak shifts to 531.6 eV because of the increasing density of electron cloud. The binding energy of O 1s returns to 532.1 eV after Z‐to‐E isomerization. Similar trends are observed for Mg 2p (the reversible shift between 50.3 and 50.7 eV). The result indicates that the solid‐state photoisomerization of PAzo‐Mg enables the reversible formation and dissociation of M—O dynamic bonds. Furthermore, owing to Mg—O dynamic‐crosslinking, PAzo‐Mg exhibits a noticeable increase in the thermal stability (5% loss at 260.7 °C) and glass transition temperature (*T*_g_ of 187.7 °C) according to thermogravimetric analysis and differential scanning calorimetry (DSC) (Figures S11 and S12, Supporting Information). Similar enhancements can also be obtained in other PAzo‐M species.

To demonstrate the effect of M—O dynamic bonds on the molecular energy level, we calculate the theoretical energy gap (∆*E*) between E‐ and Z‐PAzo‐M controlled by isomerization‐induced formation and dissociation of dynamic bonds. First, M—O dynamic bonds can form spontaneously as the Gibbs free energy (∆*G*) of all PAzo‐M species are negative (Table S2, Supporting Information). Figure 1c and Figures S13 and S14 (Supporting Information) show the optimized steric configuration of E‐PAzo‐M and Z‐PAzo‐M. As shown in Table S3 (Supporting Information), the ∆E of all PAzo‐M species increase remarkably when dynamic bonds are introduced. The formation and dissociation of dynamic bonds significantly increase the molecular energy gap by lowering the energy level of E‐isomer and increasing the energy level of Z‐isomers. This effect also potentially favors the low‐temperature reversion. ∆*E* is defined according to Equation (1) as

(1) $$\Delta E=\Delta E_{I}+\Delta E_{C}$$

∆*E*_I_ is the isomerization energy between E‐ and Z‐isomers without molecular interactions. *∆E*_C_ is the dynamic‐bond enthalpy.

Despite the variation in binding energy, it is difficult to demonstrate the relationship between M—O dynamic bonds and the increase in ∆*E*. This is because the dynamic bonds in PAzo‐M introduce the *∆E*_C_ in ∆*E* and affect the steric configuration of both E‐PAzo‐M and Z‐PAzo‐M, thereby resulting in a change of *∆E*_I_ (Figures S13 and S14, Supporting Information). Thus, the detailed relationship between the dynamic bonds and ∆E needs further investigation through experimentation. Theoretical calculations indicate that the reversible formation and dissociation of M—O dynamic bonds potentially result in a remarkable increase in energy storage capacity of PAzo‐M.

The increase in ∆*E* by using both isomerization energy and bond enthalpy of PAzo‐M is illustrated in Figure 1d. Specifically, 1) E‐PAzo‐M exhibits a lower energy level than that of E‐PAzo. E‐PAzo‐M isomerizes from the E‐ to Z‐isomer by overcoming a thermal barrier under 365 nm UV irradiation that leads to the dissociation of M—O dynamic bonds. This process enables Z‐PAzo‐M to store latent energy in chemical bonds. Z‐PAzo can store a low amount of hydrogen‐bond (H‐bond) enthalpy. 2) The metastable Z‐PAzo‐M at a high‐energy level favors the Z‐to‐E isomerization under 450 nm blue‐light irradiation, followed by the release of a large amount of isomerization energy. Z‐PAzo also undergoes a similar form of isomerization. 3) Simultaneously, PAzo‐M releases bond enthalpy by forming dynamic bonds, and H‐bond enthalpy may be released by PAzo [Equation (S1), Supporting Information]. This process indicates that energy harvesting and utilization of PAzo‐M can be optimized through isomerization and dynamic bonds.

### 2.2 Heat Storage and Release of PAzo‐Mg in Solid State

The M—O binding energy (*E*_B_) between metallic ion and organic group is an important parameter for dynamic bond. In the following section, we define the positive *E*_B_ as the absolute value of binding energy to better understand the relationship between different binding energies and photothermal properties. For dynamic polymers, the supramolecular crosslinking can be controlled by the binding energy of dynamic bonds, i.e., the crosslinking introduces a thermal barrier to the E‐to‐Z photoisomerization process. This affects the degree and kinetics of the structural transformation of azobenzenes grafted on polymers. To illustrate the relationship between the dynamic bonds and energy utilization, we track the photoisomerization [degree and kinetics, Equation (S2) and (S3), Supporting Information] using the UV–vis absorption spectra^[^ ^28^ ^]^ and measure the heat release (*E*_D_, energy density) of PAzo‐M with a variety of M—O dynamic bonds using DSC.

**Figure 2:**
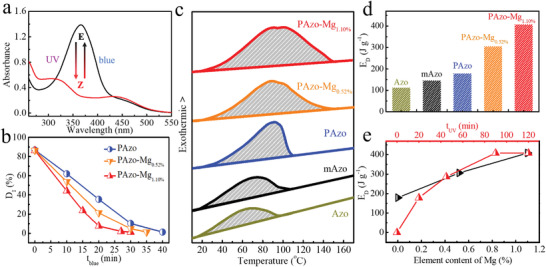
a) UV–vis absorption spectra of the PAzo‐Mg under alternate UV and blue‐light irradiation. Note: PAzo‐Mg is the PAzo‐Mg_1.10%_ in (b)–(d). b) Time‐evolved isomerization degree (*D*_I_) of PAzo, PAzo‐Mg_0.52%_, and PAzo‐Mg_1.10%_ under blue‐light irradiation. c) First heating DSC exothermic curves of Azo, mAzo, PAzo, PAzo‐Mg_0.52%_, and PAzo‐Mg_1.10%_ from 10 to 170 °C at a heating rate of 10 °C min^−1^. 0.52%, and 1.10% are the element contents of Mg. d) Histogram of the energy density (*E*_D_) of Azo, mAzo, PAzo, PAzo‐Mg_0.52%_, and PAzo‐Mg_1.10%_ after UV irradiation for 90 min. e) *E*_D_ of PAzo‐Mg as a function of the element contents of Mg in PAzo‐Mg and *t*_UV_.

We consider PAzo‐Mg as a typical dynamic polymer when compared with PAzo, and the other PAzo‐M species are also characterized for further analysis. As shown in **Figure** **2**a and Figures S15–S18 (Supporting Information), E‐PAzo‐Mg shows an intense peak at 360 nm (*π*–π* transition) and a weak broad peak at ≈450 nm (n–π* transition). After UV irradiation for 90 min (at 365 nm), there is a continuous decrease in the *π*–π* transition and a slight increase in n–π* transition due to E‐to‐Z photoisomerization. According to ^1^H NMR spectra (Figure S17, Supporting Information), the isomerization degree (*D*_I_) is 86.3% when PAzo‐Mg reaches the photostationary after 90 min. PAzo shows a similar degree of isomerization (86.8%) to that of PAzo‐Mg but a relatively high isomerization rate owing to a low thermal barrier. This result indicates that despite the crosslinking, E‐PAzo‐Mg can undergo a high degree of isomerization by overcoming the thermal barrier (isomerization energy and bond enthalpy) as long as it absorbs a sufficient number of phonons. Interestingly, the metastable Z‐PAzo‐Mg exhibits rapid Z‐to‐E transformation in the dark and under blue‐light irradiation (Figure 2b) owing to a high energy level with a first‐order rate constant (*κ*_rev_) of 1.39 × 10^−3^ s^−1^, which is higher than that for PAzo (1.16 × 10^−3^ s^−1^). This result is also confirmed by the isomerization kinetics of PAzo‐Mg, which can be controlled by the low element content of Mg. PAzo‐Mg_0.52%_ exhibits a *κ*_rev_ of 1.28 × 10^−3^ s^−1^, and a small change in *D*_I_ (Figure S18, Supporting Information). The PAzo‐Mg with a large number of dynamic bonds is PAzo‐Mg_1.10%_. 0.52% and 1.10% are the element contents of Mg obtained from the XPS spectra. When the molar ratio of Mg^2+^: Azo on PAzo further increases, the element content of Mg in PAzo‐Mg remains at 1.10% (Table S4, Supporting Information). The XPS spectra further verify the formation/dissociation of the Mg—O dynamic bonds during the above process (Figure S19, Supporting Information). The amount of Mg—O dynamic bonds determined the photothermal properties of PAzo‐M. By changing the element contents of Mg in PAzo‐Mg, we can control the amount of Mg—O dynamic bonds and further regulate its photothermal properties.

More importantly, unlike other templated azobenzene, Z‐PAzo‐Mg possesses an excellent ability to undergo a high rate of isomerization for heat release at a low temperature with a *κ*_rev_ of 1.18 × 10^−3^ s^−1^ at 4.0–5.0 °C (Figure S20, Supporting Information). This is equivalent to the *κ*_rev_ at 25.0 °C (1.39 × 10^−3^ s^−1^). Favorable isomerization at a low temperature arises from an increase in the free volume among flexible chains, which is beneficial to the low‐temperature heat output. Photoisomerization followed by the reversible formation and dissociation of M—O dynamic bonds is fundamentally important for utilizing isomerization energy and bond enthalpy. We attempted to demonstrate the effect of M—O dynamic bonds with different binding energies on heat storage and release characteristics. As seen in the DSC curves (Figure 2c), Z‐PAzo‐Mg at the photostationary can release latent heat at a relatively broad temperature range (30.0–148.0 °C) during the first heating process. As shown in Figure 2d, PAzo‐Mg_1.10%_ reaches the maximum *E*_D_ of 408.6 J g^−1^ (113.5 Wh kg^−1^), which is more than two‐fold higher than that of PAzo (179.8 J g^−1^). Compared with PAzo, mAzo, and Azo, high‐energy storage and broad exothermic temperature range of PAzo‐Mg arises from releasing both isomerization energy and Mg—O dynamic‐bond enthalpy. Additionally, PAzo exhibits a higher *E*_D_ than that of mAzo (146.8 J g^−1^), and Azo (113.6 J g^−1^) mainly by releasing isomerization energy and H‐bond enthalpy. Moreover, PAzo‐Mg exhibits a noticeable increase in its *E*_D_ values with the increasing element content of Mg (Figures S21 and S22, Supporting Information) and long UV irradiation time (*t*_UV_) (Figure 2e; Figures S23 and S24, Supporting Information). For instance, *t*_UV_ of 120 min, the balance in *D*_I_ (86.5%) results in a high *E*_D_ (408.0 J g^−1^), and PAzo‐Mg_0.52%_ only shows an *E*_D_ of 306.6 J g^−1^. The tunable *E*_D_ of PAzo‐Mg reveals that the heat release is attributed to that induced by photoisomerization and the corresponding formation of Mg—O dynamic bonds instead of the photothermal effect. This result indicates that heat storage and release of PAzo‐Mg is increased by the Mg—O dynamic bonds that offer a solid foundation to further analyze the effect of the dynamic bonds on energy utilization.

### 2.3 Influence of Metal‐Group Species on Photoisomerization and Thermal Energy Release

**Figure 3:**
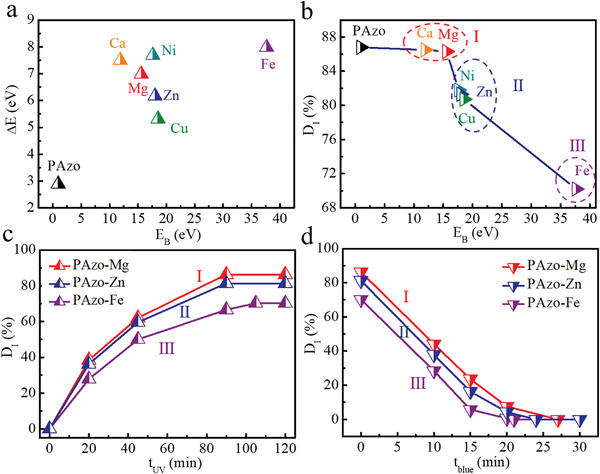
a) Δ*E* and b) *D*_I_ of PAzo‐M (M = Mg, Ca, Ni, Zn, Cu, Fe) and PAzo controlled by M—O dynamic bonds with different *E*_B_ values. Time‐evolved *D*_I_ of (I) PAzo‐Mg, (II) PAzo‐Zn, and (III) PAzo‐Fe along with c) UV irradiation time (*t*_UV_) and d) blue‐light irradiation time (*t*_blue_).

The formation and dissociation of M—O dynamic bonds not only change molecular levels of azobenzene on the polymers but also affect the isomerization due to crosslinking. The combined effect results in an increase in heat storage and release. The theoretical Δ*E* of PAzo‐M controlled by M—O dynamic bonds with different *E*_B_ values is summarized in **Figure** **3**a, Tables S3 and S5 (Supporting Information). PAzo‐Ca (7.49 eV), PAzo‐Mg (6.98 eV), PAzo‐Ni (7.69 eV), and PAzo‐Fe (7.98 eV) for a wide range of *E*_B_ exhibit higher Δ*E* values than those of PAzo‐Zn (6.16 eV) and PAzo‐Cu (6.31 eV). This is because the theoretical Δ*E* is mainly determined by the molecular level of E‐PAzo‐M and Z‐PAzo‐M, and the degree and kinetics of photoisomerization are ignored. A high heat storage capacity of PAzo‐M species include high theoretical Δ*E* and experimental *D*_I_.^[^ ^29^ ^]^

In contrast, Figure 3b shows a large difference in the *D*_I_ of PAzo‐M controlled by M—O dynamic bonds with different *E*_B_ values. For an in‐depth understanding, we divide PAzo‐M into three categories: (I) PAzo‐Mg and PAzo‐Ca with a low *E*_B_ < 16 eV; (II) PAzo‐Ni, PAzo‐Cu, and PAzo‐Zn with a moderate *E*_B_ in the range of 16–20 eV; and (III) PAzo‐Fe with a high *E*_B_ > 30 eV. When *E*_B_ increases, a noticeable decrease occurs in the *D*_I_ of PAzo‐M with a relatively low rate of isomerization (Figure 3c and Figure S25a, Supporting Information). This indicates that a high *E*_B_ leads to strong crosslinking by M—O dynamic bonds, which limits the isomerization because of the introduction of a high thermal barrier for the E‐to‐Z isomerization. For example, PAzo‐M in (I) and (II) maintains a relatively high *D*_I_ of 80.0%–86.0%, but PAzo‐Fe exhibits a significantly low *D*_I_ of 70.2%, which inevitably lowers the experimental heat storage.

PAzo‐Fe (III) also exhibits the lowest rate of isomerization with the highest *E*_B_ and thermal barrier. However, it also has a higher *κ*_rev_ of 2.45 × 10^−3^ s^−1^ compared with those of PAzo‐Mg (I, 1.39 × 10^−3^ s^−1^) and PAzo‐Zn (II, 1.72 × 10^−3^ s^−1^) during Z‐to‐E isomerization under blue‐light irradiation (Figure 3d; Figures S25 and S26, Supporting Information). This may be owing to the fact that Z‐PAzo‐Fe exhibits the highest energy level and needs to overcome the low energy barrier for Z‐to‐E reversion. This result indicates that based on a high theoretical Δ*E*, M—O dynamic bonds with a suitable *E*_B_ favors photoisomerization for inducing the formation and dissociation of dynamic bonds owing to mild crosslinking.

### 2.4 Mechanism for Energy Level Regulation between E‐ and Z‐Isomer

**Figure 4:**
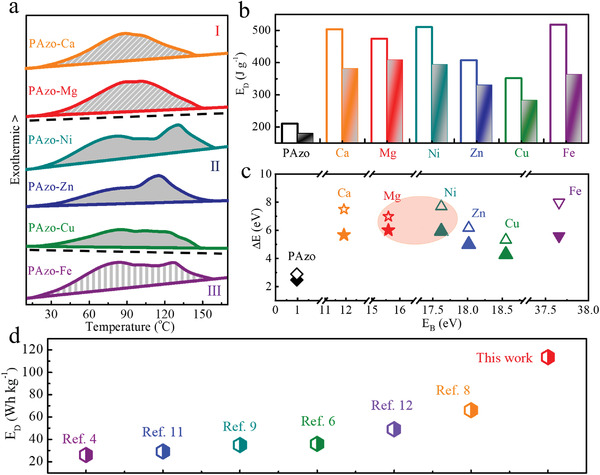
a) Exothermic DSC curves of PAzo‐M at a heating rate of 10 °C min^−1^. b) Histogram of the theoretical (hollow) and experimental (solid) *E*_D_ of PAzo and PAzo‐M; c) theoretical (hollow, ◇, △, ▽, ☆) and experimental (solid, ◆, ▲, ▼, ★) ∆*E* of PAzo and PAzo‐M with M—O dynamic bonds of different *E*_B_ values. d) Comparison of *E*_D_ for different polymer‐templated azobenzene.

We measure the heat release of a variety of PAzo‐M species using DSC to demonstrate the relationship between M—O dynamic bonds and the utilization of both isomerization energy and bond enthalpy. As shown in **Figure** **4**a,b and Table S6 (Supporting Information), all the PAzo‐M species can release both isomerization energy and bond enthalpy with a significantly high *E*_D_ of 283.0–408.6 J g^−1^ (78.6–113.5 Wh kg^−1^) at a temperature range of 25.0–175.0 °C, which outperforms all previous azo‐based polymers.^[^ ^10^, ^11^, ^12^ ^]^ The *E*_D_ of PAzo‐M can be controlled by dynamic bonds. However, the experimental *E*_D_ is different from theoretical calculation *E*_D_ due to the difference of *D*_I_ (Figure 4b). Interestingly, PAzo‐Mg in (I) shows a maximum *E*_D_ of 408.6 J g^−1^ (113.5 Wh kg^−1^), which is close to that of PAzo‐Ni (394.2 J g^−1^) in the (II). The *E*_D_ of PAzo‐Mg is 7.1%–12.5% higher than those of PAzo‐Ca (I, 381.4 J g^−1^) and PAzo‐Fe (III, 363.3 J g^−1^), and considerably higher (23.7%–44.4%) than those of PAzo‐Zn (330.3 J g^−1^) and PAzo‐Cu (283.0 J g^−1^) in (II). Additionally, the exothermic PAzo‐M in (I) is prone to releasing heat at a relatively low temperature with only one broad exothermic peak. In contrast, with a relatively high *E*_B_, PAzo‐M in (II) and (III) exhibit two exothermic peaks at different temperatures, indicating a stepwise release of isomerization energy (at a low temperature range of 60–100 °C), followed by dynamic‐bond enthalpy (at a high‐temperature range of 100–150 °C). Furthermore, the number of dynamic bonds is another parameter that affects energy storage. Although PAzo‐Ca (I) exhibits a higher ∆*E* (7.49 eV) and a comparable *D*_I_ (86.6%) compared with PAzo‐Mg, it exhibits an *E*_D_ of 381.4 J g^−1^ owing to the low element content of Ca (0.89%). This analysis is confirmed by the exothermic peak of PAzo‐Ca, which is similar to that of PAzo‐Mg in (I) (Figure 4a).

According to Equation (2)

(2) $$E_{D}=\Delta E\times D_{I}$$

The ability of PAzo‐M to harvest light and release heat is determined by the combination of a large ∆*E* and high *D*_I_. In other word, PAzo‐M can store and release high isomerization energy and bond enthalpy based on favorable isomerization for the formation and dissociation of high‐energy M—O dynamic bonds. For PAzo‐Mg (low *E*_B_) in (I), high‐energy storage is mainly attributed to a favorable isomerization with a significantly high *D*_I_ (86.3%) and a relatively high ∆*E* (6.98 eV), whereas PAzo‐Ni in (II) relies on a large energy difference (∆*E* = 7.69 eV) with a slightly reduced *D*_I_ (81.8%). Moreover, the two exothermic peaks of PAzo‐Ni arising from the hysteretic release of bond enthalpy indicates a low rate of Z‐to‐E isomerization at the same temperature. In contrast, an obvious decrease in the *E*_D_ of PAzo‐Zn (Cu) and PAzo‐Fe is caused by a significantly low energy storage capacity (∆*E* of 6.16 and 5.31 eV) and a limited isomerization (*D*_I_ of 70.2%), respectively.

The M—O dynamic bonds in PAzo‐M can be designed and used to cross‐link polymer chains as a thermal barrier to isomerization and increase the energy density by introducing bond enthalpy. The crosslinking and steric hindrance can favor or limit photoisomerization and the corresponding formation and dissociation of dynamic bonds. Thus, in general, an effective strategy to increase the *E*_D_ of PAzo‐M is to selectively optimize the M—O dynamic bonds to increase ∆*E* and *D*_I_ based on suitable crosslinking and molecular energy levels. As a result, the suitable *E*_B_ of Mg—O dynamic bonds enables PAzo‐Mg in (I) to isomerize to high‐energy metastable states (∆*E* of 6.98 eV) at a high *D*_I_ of 86.3%, thereby resulting in a maximum *E*_D_ of 408.6 J g^−1^ with an excellent power density (Figure S26d, Supporting Information). This result indicates that two interrelated properties, including a high ∆*E* due to the molecular design and favorable isomerization due to weakened crosslinking, are of great importance for light‐harvesting and the utilization of isomerization energy and bond enthalpy (Figure 4c). Additionally, PAzo‐Mg exhibits an excellent *E*_D_ (Figure 4d) when compared with related polymer‐templated azobenzene of different molecular structures. PAzo‐Mg possesses the ability to harvest and use high energy and can isomerize rapidly at low temperatures. Therefore, it is an ideal photoresponsive candidate for flexible self‐heated textiles.

### 2.5 Flexible and Wearable Thermal Management Device: Applications Based on the Photodynamic Bonds of PAzo‐Mg

**Figure 5:**
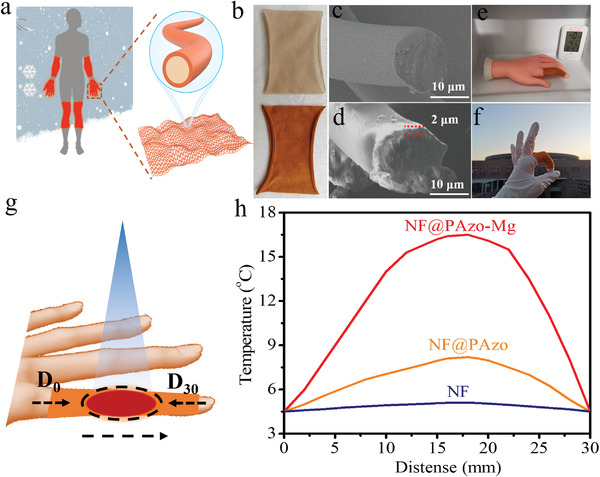
a) Schematic and b) optical image of the NF@PAzo‐Mg textile. c,d) SEM images of the cross section of NF and NF@PAzo‐Mg with a coating of 2 µm thickness. Image of NF@PAzo‐Mg e) on model hands (indoors) at 0.0–4.0 °C and f) real hands (outdoors) at −5.0 to −3.0 °C. g) Schematic of the heat release area of NF@PAzo‐Mg. The temperatures within *D*_0_–*D*_30_ (where the subscript denotes the distance from *D*_0_ in mm) were tracked. h) Temperatures from *D*_0_ to *D*_30_ in the area with NF@PAzo‐Mg, NF@PAzo, and NF.

The self‐heating performance of a flexible textile is investigated after coating PAzo‐Mg on a nylon fabric (NF) to form NF@PAzo‐Mg. Uniform coating on the textile is a prerequisite for the long‐term thermal management of wearable devices (**Figure** **5**a). Unlike mAzo and PAzo powders, PAzo‐Mg can form a self‐supporting film with the smooth surface (Figures S27 and S28, Supporting Information). As shown in Figure 5b–d, the PAzo‐Mg layer with a thickness of ≈2 µm (cross‐section scanning electron microscope [SEM] image in Figure 5d) is uniformly assembled on the surface of an individual nylon fiber using a 5–10 mg mL^−1^ PAzo‐Mg solution. Intermolecular H‐bonds between PAzo‐Mg and the polyamides enable PAzo‐Mg to be coated on the nylon fibers with strong adhesion instead of forming an aggregate on the fibers or uniformly dispersing in the gaps of the fabric. The concentration of PAzo‐Mg solution is used to maintain the uniformity of coating (Figure S29, Supporting Information). Thus, a suitable thickness enables PAzo‐Mg to uniformly adhere to the surface of the fibers, and favors light harvesting for a high degree of isomerization. The flexible NF@PAzo‐Mg provides a tensile strength of 0.42 MPa with a high strain of 90%, and it also shows an elastic deformation after 100 cycles (Figure S30, Supporting Information). The elasticity originates from the stretching of the weaving structure. The flexible NF@PAzo‐Mg is suitable for assembly in a smart wearable device.

### 2.6 Heat Release of NF@PAzo‐Mg Wearable Thermal Management Device Triggered by Light in Cold Environment for Keeping Warm

Previous studies focused on the heat release of a film based on photothermal conversion at a relatively high temperature (>25.0 °C). However, the difficulty in assembly and high‐temperature heat release limits the future application in smart wearable devices. Currently, a great challenge for flexible self‐heated textiles is the control of heat release at a low temperature based on the cyclic utilization of light energy without a power supply. The flexible NF@PAzo‐Mg is tailored to be worn on the model and real hands at −5.0 to 5.0 °C placed indoors and outdoors for self‐heating, respectively.

**Figure 6:**
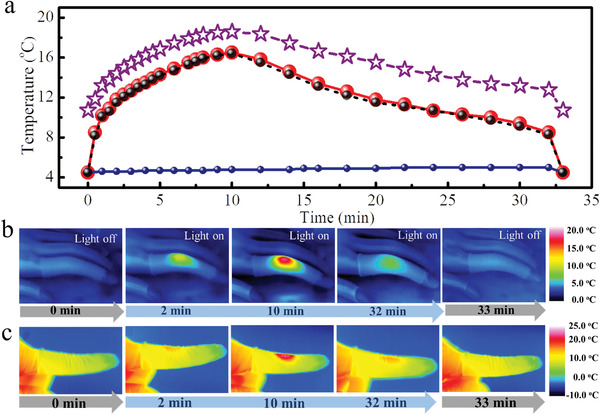
a) Time‐evolved temperature of NF (blue, ●), NF@PAzo‐Mg (red, ●), and NF@PAzo‐Mg (black, ●) at a strain of 50% on model hands (indoors) and NF@PAzo‐Mg (purple, ☆) on real hands (outdoors) under blue‐light irradiation for 32 min. The temperature–time curves are drawn by the temperature of the irradiation center. Corresponding infrared (IR) thermal images of NF@PAzo‐Mg b) on model hands (indoors) at an ambient temperature of 0.0–4.0 °C and c) real hand (outdoors) at an ambient temperature of −5.0 to −3.0 °C.

We investigate the low‐temperature heat release of NF@PAzo‐Mg using three steps: (i) light harvesting by absorbing UV light (80 mW cm^−2^) for 90 min (*D*_I_ = 86.3%); (ii) storage at −5.0 to 5.0 °C for 1 h (energy loss of ≈0.1% according to Figure S31, Supporting Information); (iii) heat release induced by blue‐light irradiation with 40 mW cm^−2^ for 32 min. During step (iii), we track the time‐evolved temperature change of two NF@PAzo‐Mg fabrics on different hands (indoor and outdoor) using a high‐resolution IR camera. The model hand (with dimensions of 30 cm × 10 cm × 8 cm) with the NF@PAzo‐Mg textile was placed in a refrigerator at 0–4.5 °C (Figure 5e and Figure S32, Supporting Information). With the irradiation of blue light, the textile (4.5 °C) can release heat (both isomerization energy and bond enthalpy) to induce a continuous increase in temperature (**Figure** **6**a,b).

The temperature reaches a maximum of 16.5 °C (Figure 5h) with a ∆*T* (difference between maximum temperature and ambient temperature) of 12.0 °C after 10 min owing to a rapid Z‐to‐E isomerization. There exists an equilibrium between self‐heating and thermal exchange with the environment. Next, the temperature of textile gradually decreases to 8.5 °C within the next 22 min owing to a lower rate of heat output than heat loss. Then, the temperature of the textile quickly returns to 4.5 °C when the irradiation is terminated. The self‐heated textile displays excellent cycling performance (Figure S33, Supporting Information) after ten cycles with a fluctuation of ∆*T* (11.5–12.0 °C). Furthermore, the NF@PAzo‐Mg textile at a 50% tensile strain has almost the same temperature curve, thereby indicating that the textile at this potential tensile strain can provide heat continuously during human activity. Additionally, the NF@PAzo textile shows a ∆*T* of 6.3 °C (Figure S32c, Supporting Information). NF@PAzo‐Mg and the NF textile without light‐harvesting exhibit a ∆*T* of 4.0 and 0.5 °C under the same condition owing to photothermal conversion.

Furthermore, we demonstrate the self‐heated performance of the flexible NF@PAzo‐Mg textile on a human wearing it as a self‐heated glove outdoors (temperature of −5.0 to −3.0 °C, humidity of 10.0% and wind speed of 5.5–7.9 m s^−1^). Despite a low ambient temperature, the starting temperature of the textile on one finger is 10.8 °C owing to the body heat of the human (Figure 6a and Figure S34, Supporting Information). Under blue‐light irradiation, the NF@PAzo‐Mg textile exhibits an increasing temperature from 10.8 to 18.5 °C with a ∆*T* of 7.7 °C in 10 min and a decrease in temperature in the next 22 min (Figure 6c). Unlike that for the textile on the model hand, the reduced ∆*T* for the textile on the human hand is attributed to a large amount of heat loss by air cooling. The temperature of NF@PAzo‐Mg textile also shows a clear rising gradient on the model and real hands, and the temperature decreases when it is far from the irradiation center owing to a low degree of isomerization followed by the formation of a few Mg—O dynamic bonds. This feature demonstrates potential for self‐heating by controlling the irradiation range and power density of blue light. Meanwhile, it also enables the NF@PAzo‐Mg textile to selectively improve the increase in the temperature at a specific region by self‐heating according to the change in ambient temperature.

## 3 Conclusion

We demonstrate the co‐harvesting of isomerization energy and bond enthalpy of a series of photoresponsive PAzo‐M species by light absorption, low‐temperature storage, and heat release. Introducing dynamic bonds into crosslinked PAzo‐M species increases the ∆*E* and lowers the thermal barrier for Z‐to‐E isomerization owing to photocontrolled reversible formation and dissociation. By choosing a suitable metallic ion for the M—O dynamic bond, suitable binding energy can be achieved, favoring a rapid and high degree of photoisomerization at low temperatures and releasing a large amount of photothermal energy and bond enthalpy. PAzo‐Mg exhibits a maximum energy density of 408.6 J g^−1^ (113.5 Wh kg^−1^) with a relatively high Δ*E* (6.98 eV) and *D*_I_ (86.3%). We also fabricate a wearable self‐heated textile coated with PAzo‐Mg to realize temperature control in low‐temperature environments. A uniform NF@PAzo‐Mg textile can selectively release heat for a ∆*T* of 7.7–12.0 °C at a low temperature of −5.0 to 5.0 °C. This new strategy for tuning the heat release of photothermal polymers by controlling isomerization‐induced formation and dissociation of photodynamic bonds provides an avenue for self‐heated wearable thermal management devices, smart on‐skin clothing, and soft robotics applications.

## Conflict of Interest

The authors declare no conflict of interest.

## Supporting information

Supporting Information

## Data Availability

Research data are not shared.

## References

[1] B. Yang , F. Cai , S. Huang , H. Yu , Angew. Chem., Int. Ed. 2020, 59, 4035. 10.1002/anie.201914201 31823474

[2] L. Fei , Y. Yin , M. Yang , S. Zhang , C. Wang , Energy Storage Mater. 2021, 42, 636.

[3] J. Xiong , J. Chen , P. S. Lee , Adv. Mater. 2021, 33, 2002640. 10.1002/adma.202002640 PMC11468729 33025662

[4] D. Zhitomirsky , J. C. Grossman , ACS Appl. Mater. Interfaces 2016, 8, 26319. 27611884 10.1021/acsami.6b08034

[5] J. Hu , S. Huang , M. Yu , H. Yu , Adv. Energy Mater. 2019, 9, 1901363.

[6] K. Ishiba , M. Morikawa , C. Chikara , T. Yamada , K. Iwase , M. Kawakita , N. Kimizuka , Angew. Chem., Int. Ed. 2015, 54, 1532. 10.1002/anie.201410184 25483773

[7] M. A. Gerkman , R. S. Gibson , J. Calbo , Y. Shi , M. J. Fuchter , G. G. Han , J. Am. Chem. Soc. 2020, 142, 8688. 32319773 10.1021/jacs.0c00374

[8] G. D. Han , S. S. Park , Y. Liu , D. Zhitomirsky , E. Cho , M. Dincă , J. C. Grossman , J. Mater. Chem. A 2016, 4, 16157.

[9] A. K. Saydjari , P. Weis , S. Wu , Adv. Energy Mater. 2017, 7, 1601622.

[10] H. Zhou , C. Xue , P. Weis , Y. Suzuki , S. Huang , K. Koynov , G. K. Auernhammer , R. Berger , H. Butt , S. Wu , Nat. Chem. 2017, 9, 145. 28282043 10.1038/nchem.2625

[11] D. Zhitomirsky , E. Cho , J. C. Grossman , Adv. Energy Mater. 2016, 6, 1502006.

[12] L. Fu , J. Yang , L. Dong , H. Yu , Q. Yan , F. Zhao , F. Zhai , Y. Xu , Y. Dang , W. Hu , Y. Feng , W. Feng , Macromolecules 2019, 52, 4222.

[13] L. Zhao , Y. Liu , R. Xing , X. Yan , Angew. Chem., Int. Ed. 2020, 59, 3793. 10.1002/anie.201909825 31571353

[14] O. Vybornyi , S. X. Liu , R. Häner , Angew. Chem., Int. Ed. 2021, 60, 25872. 10.1002/anie.202108745 PMC9298031 34529324

[15] Z. Liu , F. Yan , Adv. Sci. 2022, 2200264. 10.1002/advs.202200264 PMC9036041 35233988

[16] D. Su , S. Zhou , H. Masai , Z. Liu , C. Zhou , C. Yang , Z. Li , S. Tsuda , Z. Liu , J. Terao , X. Guo , Adv. Sci. 2022, 2200022. 10.1002/advs.202200022 PMC9069358 35233985

[17] R. Klajn , Chem. Soc. Rev. 2014, 43, 148. 23979515 10.1039/c3cs60181a

[18] L. Dong , Y. Feng , L. Wang , W. Feng , Chem. Soc. Rev. 2018, 47, 7339. 30168543 10.1039/c8cs00470f

[19] L. You , D. Zha , E. Anslyn , Chem. Rev. 2015, 115, 7840. 25719867 10.1021/cr5005524

[20] Y. Ma , P. She , K. Zhang , H. Yang , Y. Qin , Z. Xu , S. Liu , Q. Zhao , W. Huang , Nat. Commun. 2018, 9, 3. 29317626 10.1038/s41467-017-02452-w PMC5760713

[21] A. Khayyami , A. Philip , M. Karppinen , Angew. Chem., Int. Ed. 2019, 58, 13400. 10.1002/anie.201908164 31318130

[22] C. Li , J. Zuo , Adv. Mater. 2020, 32, 1903762. 10.1002/adma.201903762 31599045

[23] R. Pearson , J. Am. Chem. Soc. 1963, 85, 3533.

[24] J. Liu , C. Xie , A. Kretzschmann , K. Koynov , H. Butt , S. Wu , Adv. Mater. 2020, 32, 1908324. 10.1002/adma.201908324 32091153

[25] J. Han , C. Xie , Y. Huang , M. Wagner , W. Liu , X. Zeng , J. Liu , S. Sun , K. Koynov , H. Butt , S. Wu , J. Am. Chem. Soc. 2021, 143, 12736. 34346213 10.1021/jacs.1c05648

[26] T. J. Kucharski , N. Ferralis , A. M. Kolpak , J. O. Zheng , D. G. Nocera , J. C. Grossman , Nat. Chem. 2014, 6, 441. 24755597 10.1038/nchem.1918

[27] X. Zhang , L. Hou , P. Samorì , Nat. Commun. 2016, 7, 11118. 27067387 10.1038/ncomms11118 PMC4832057

[28] J. Liu , S. Wang , T. Huang , P. Manchanda , E. Abou‐Hamad , S. Nunes , Sci. Adv. 2020, 6, eabb3188. 32875111 10.1126/sciadv.abb3188 PMC7438094

[29] T. Kucharski , Y. Tian , S. Akbulatova , R. Boulatov , Energy Environ. Sci. 2011, 4, 4449.

