# Supplementary material for: Metallic‐Ion Controlled Dynamic Bonds to Co‐Harvest Isomerization Energy and Bond Enthalpy for High‐Energy Output of Flexible Self‐Heated Textile
Source: Adv Sci (Weinh). 2022 May 1;9(20):2201657. doi: 10.1002/advs.202201657 (PMC9284279; doi:10.1002/advs.202201657)
Supplement: Supplementary file 1 — Supporting Information [file ADVS-9-2201657-s001.pdf]

ADVANCED SCIENCE
Open Access

Supporting Information

for *Adv. Sci.*, DOI 10.1002/advs.202201657

Metallic-Ion Controlled Dynamic Bonds to Co-Harvest Isomerization Energy and Bond Enthalpy for High-Energy Output of Flexible Self-Heated Textile

Hui Wang, Yiyu Feng, Jian Gao, Wenyu Fang, Jing Ge, Xiaoyu Yang, Fei Zhai, Yunfei Yu and Wei Feng**

Supporting Information

Metallic-ion Controlled Dynamic Bonds to Co-harvest Isomerization Energy and Bond Enthalpy for High-Energy Output of Flexible Self-Heated Textile

Hui Wang,[a] *Yiyu Feng,**[a,b] *Jian Gao,*[a] *Wenyu Fang,*[a] *Jing Ge,*[a] *Xiaoyu Yang,*[a] *Fei Zhai,*[a] *Yunfei Yu,*[a] *and Wei Feng**[a]

[a] H. Wang, Prof. Y. Feng, J. Gao, W. Fang, J. Ge, X. Yang, F. Zhai, Y. Yu, Prof. W. Feng

School of Materials Science and Engineering, Tianjin University, Tianjin 300350, China

* Corresponding Author E-mail: fengyiyu@tju.edu.cn (Y. Feng), weifeng@tju.edu.cn (W. Feng)

[b] Prof. Y. Feng

Key Laboratory of Materials Processing and Mold, Ministry of Education, Zhengzhou University, Zhengzhou 450002, Henan, China

Table of Contents

1. Experimental Section 3

1.1 Methods 3

1.2 Characterization 4

1.3 Flexible and wearable thermal management device 6

2. Supplementary Figures 7

3. Supplementary Tables 26

4. Supplementary Equations 30

5. References 31

1. Experimental Section

1.1 Methods

1.1.1 Materials

The 4'-aminoazobenzene-4-sulphonic acid (CAS: 104-23-4) used in this study was a commercially available reagent purchased from Bide Pharmatech Ltd. and used as received. Acryloyl chloride (98.0%), tetrahydrofuran (THF, ≥99.8%, safe dry, water ≤50 ppm), triethylamine (99.9%), dichloromethane (DCM, ≥99.8%, safe dry, water ≤50 ppm), azobisisobutyronitrile (AIBN, 99.0%), methanol (≥99.8%, safe dry, water ≤100 ppm), dimethyl sulfoxide (DMSO, ≥99.7%, safe dry, water ≤50 ppm), ethanol (≥99.5%, safe dry, water ≤50 ppm) were purchased from Adamas. Magnesium chloride ($MgCl_2$, safe dry, 99.99% metals basis), iron (III) chloride ($FeCl_3$, safe dry, 99.99% metals basis), copper chloride ($CuCl_2$, safe dry, 99.99% metals basis) were purchased from Aladdin. Calcium chloride anhydrous ($CaCl_2$, safe dry, 97.0% metals basis) was obtained from Sigma-Aldrich. Nickel chloride ($NiCl_2$, safe dry, 99.99% metals basis), and zinc chloride ($ZnCl_2$, safe dry, 99.95% metals basis) were obtained from Alfa. All reagents were used directly without further purification.

1.1.2 Synthesis of the azobenzene monomer (mAzo)

The monomer was synthesized according to a published recipe.[1] Briefly, 4'-aminoazobenzene-4-sulphonic acid (9.0 mmol) was dissolved in dry tetrahydrofuran (THF, 100 mL) and triethylamine (2.5 mL) under anhydrous and oxygen free conditions. Acryloyl chloride (1.0 mL, 13.0 mmol) was added dropwise under inert conditions during the cooling reaction in an ice bath for 4 h. Subsequently, the mixture was stirred at 25 ^oC for 48 h. The monomer was extracted by diluting it three times with a 3:1 mixture of dichloromethane (DCM) and H_2O. The organic phase was dried with sodium sulfate and dried in a vacuum (<0.1 mbar) overnight. The resultant material was further purified in a silica column using a 1:1 ratio of DCM and hexanes. The overall reaction yield was between 50% and 60%. ^1H NMR (DMSO-d6): δ (ppm) = 7.90, 7.90–7.74, 7.74–7.62 (protons on the azobenzene group), 5.82 (dd, 1H, -CH=CH_2), 6.3 (dd, 1H, -CH=CH_2), 6.48 (dd, 1H, -CH=CH_2).

1.1.3 Synthesis of the azobenzene polymer (PAzo)

Polymer-templated azobenzene (PAzo) was synthesized by free radical polymerization of mAzo in THF using azobisisobutyronitrile (AIBN) as the initiator. First, in the typical homopolymerization stage, the monomer (1.0 g) was dissolved in anhydrous THF (20 mL) and AIBN (30 mg) was added. The solution was subjected to three freeze/pump/thaw cycles. The reaction was run under inert conditions at 60 °C for 12 h. The mixture was concentrated and then poured into methanol and the yellow precipitate was isolated. The precipitate was filtered and rinsed with additional methanol, and the polymer PAzo was obtained. The maximum yields for the reaction were 60–80%. ^1H NMR (DMSO-d6): δ (ppm) = 7.91, 7.85–7.76, 7.69 (protons on the azobenzene group), 1.5–1.0 (protons on the polymer backbone).

1.1.4 Synthesis of the photodynamic azobenzene polymer (PAzo-M)

50 mg PAzo was ultrasonically dissolved in 20 mL anhydrous DCM: ethanol (1:1). An appropriate amount of MCl_n ($MgCl_2$, $CaCl_2$, $NiCl_2$, $ZnCl_2$, $CuCl_2$, $FeCl_3$) was

dissolved in 2 mL anhydrous ethanol at molar ratio of 1:1 (MCl_n: Azo on PAzo). Subsequently, the MCl_n solution was added to the PAzo solution and ultrasound for 2 min to make the mixture uniform. Then, PAzo and MCl_n rapidly formed precipitates (PAzo-M). After 1 h of reaction, the precipitates were taken out and washed with anhydrous ethanol several times to remove the remaining unprecipitated PAzo in the upper dispersion and excessive MCl_n. Then, the above precipitates were dried in a vacuum overnight at 80.0 ºC/24 h to obtain the photodynamic azobenzene polymer (PAzo-M). The above synthesis steps were completed in an Ar-filled glove box to avoid the influence of H_2O.

To explore the effects of different element contents of Mg in PAzo-Mg on photothermal properties, molar ratios of 0:1, 1:4, and 1:1 of $MgCl_2$: Azo on PAzo were tested. These were denoted as $PAzo_{0.0\%}$, PAzo-$Mg_{0.52\%}$, and PAzo-$Mg_{1.10\%}$, where 0.0%, 0.52%, 1.10% represent Mg element content in the PAzo-Mg samples based on XPS. When the molar ratio of Mg^{2+}: PAzo further increases, the element content of Mg in PAzo-Mg remains at 1.10%. Note: PAzo-$Mg_{1.10\%}$ is the PAzo-Mg discussed in the article. ^{1}H NMR (DMSO-d6) of PAzo-Mg: δ (ppm) = 7.92, 7.89–7.75, 7.70 (protons on the azobenzene group), 1.5–1.0 (protons on the polymer backbone). Metal photodynamic interaction has little effect on the ^{1}H NMR results of PAzo owing to a small metal element content.

1.1.5 Preparation of the wearable thermal management device-photothermal fabric (NF@PAzo-Mg)

A commercial nylon fabric made up of 100% polyamide was purchased from the local market. The commercial cloth was used as a template without special treatment. The nylon fabric (NF) was immersed in a 10 mg mL^{-1} DMSO solution of PAzo-Mg for 5 min and dried in vacuum overnight at 100.0 ºC/48 h to remove the DMSO, thereby resulting in a flexible and wearable thermal management device-photothermal fabric (NF@PAzo-Mg). The uniform PAzo-Mg functional layer was assembled on the surface of nylon fabric fiber through hydrogen bonding instead of dispersed in the gaps of the fabric texture. To optimize the thickness of the PAzo-Mg coating layer, the nylon fabric was immersed in 1 mg mL^{-1}, 10 mg mL^{-1}, and 20 mg mL^{-1} PAzo-Mg solutions to obtain NF@PAzo-Mg, respectively. The above synthesis steps were completed in an Ar-filled glove box to avoid the influence of H_2O.

1.2 Characterization

1.2.1 Computational methods.

The single-point energies of the cis- and trans- forms of PAzo and PAzo-M (M = Mg, Ca, Fe, Ni, Cu and Zn) were calculated using density functional theory (DFT). The DFT calculations were performed using Gaussian16 at the B3LYP/6-31G(d) level with the Grimme’s D3(BJ) empirical dispersion correction.

1.2.2 Morphology and structure characterization

The morphology of the nylon fabric and NF@PAzo-Mg was observed using a scanning electron microscope (SEM; S-4800, Hitachi, Japan). The chemical structure of the PAzo-Mg, PAzo, mAzo, and Azo was analyzed using Fourier transform infrared (FTIR) spectra recorded on a Tensor 27 spectrometer (Bruker, Germany) based on pure KBr discs. X-ray photoelectron spectroscopy (XPS) analysis was performed on a

surface element analysis system (Axis Supra, Kratos, Japan). Thermal analysis was performed using a thermogravimetric analyzer (TG/DTA; STA449f3, NETZSCH, Germany) protected by 50 mL min^{-1} nitrogen purging at a heating rate of 10 °C min^{-1} from 30 to 800 °C. Time-evolved UV-Vis absorption spectra were obtained using a UV-Vis spectrophotometer (UV-3600 Plus, Shimadzu, Japan) in a 10 mm pathlength quartz cuvette. The heat flow of the charged samples was evaluated using DSC analysis with a Q series Q20 instrument (DSC; Q20, TA Instruments, USA). ^{1}H NMR spectra were obtained using a 400-MHz spectrometer (INOVA, Varian) with trimethylsilyl as an internal standard. The molecular weights and polydispersity indices of the PAzo were determined by gel permeation chromatography (GPC) (Agilent PL-GPC50). DMSO was used as the eluent and polystyrene was used as a standard. The exothermic temperature of NF@PAzo-Mg and nylon fabric under blue-light irradiation was tracked using a high-resolution infrared (IR) thermal imaging camera (Fluke TiX640 Expert HD, USA).

1.2.3 Trans (E) to cis (Z) isomerization (charging)

For solid-state trans to cis (E-to-Z) isomerisation, PAzo-M (M = Mg, Ca, Ni, Zn, Cu, and Fe) and NF@PAzo-Mg were charged under controlled UV irradiation for different times (HTLD-4II, Shenzhen Height-LED Opto-electronics Tech Co., Ltd., China). The light intensity was measured with a full-spectrum optical power metre (CEL-NP2000-10, Beijing China Education Au-light Co., Ltd.). The solid samples were transferred to glass substrates and irradiated by a UV lamp (365 nm, 80 mW cm^{-2}) placed 5 cm above at room temperature (25.0 °C). After 90 min of irradiation, the samples were stored in the dark for further measurements. The UV charging station was covered with a container and an aluminium foil to block ambient light exposure.

1.2.4 Cis (Z) to trans (E) isomerization (discharging)

For solid-state cis to trans (Z-to-E) reversion, PAzo-M (M = Mg, Ca, Ni, Zn, Cu, and Fe) and NF@PAzo-Mg after 90 min UV irradiation were irradiated under controlled blue light (450 nm, 40 mW cm^{-2}) placed 5 cm above for different times at 25.0 °C.

The heat flow of the charged samples was evaluated using DSC analysis with a Q series Q20 instrument (DSC; Q20, TA Instruments, USA). The charged samples were transferred to a hermetically sealed DSC pan. All experiments were performed at a scan rate of 10 °C min^{-1} and in the scan temperature region of 10-170 °C except for the experiments conducted to check for a possible relationship between the temperature elevation rates and exothermic values. The heat storage density obtained by DSC was calculated based on the first scan. There was no heat flow in the second heat cycle, indicating that the heat in the first cycle was released from the PAzo-M after storing solar energy. The PAzo-M could be discharged via blue-light irradiation or heating.

For comparison, PAzo and mAzo powder samples were also subjected to the same charging/discharging experiments.

The E-to-Z isomerisation process and Z-to-E reversion process were investigated using ^{1}H NMR and a UV-Vis spectrophotometer. The samples tested with different

isomerisation degrees were redispersed in DMSO. Next, they were covered with aluminium foil in the dark or irradiated with a 365 UV lamp or 450 nm visible light at room temperature for different times. The rate of reversion was calculated based on the intensity change of the transition bands of PAzo and PAzo-Mg from the UV-Vis absorption spectra. The isomerisation degree (D_I) of the metastable state (cis-rich) was measured to be 86.3% for PAzo-Mg and 86.8% for PAzo based on ^{1}H NMR spectra and the stable state (trans-rich) was ~100% trans isomer.

1.3 Flexible and wearable thermal management device

We investigated the low-temperature heat release of NF@PAzo-Mg by three steps.

(i) Light harvesting by absorbing 365 nm UV light (80 mW cm^{-2}) for 90 min (D_I = 86.3%);

(ii) Storage at -5.0–5.0 °C for 1 h (energy loss of approximately 0.1% according to **Figure S31**);

(iii) Heat release induced by 450 nm blue-light irradiation with 40 mW cm^{-2} for 32 min. During step (iii), we tracked the time-evolved temperature change of two NF@PAzo-Mg on different hands using a high-resolution infrared thermal imaging camera (Fluke TiX640 Expert HD, USA).

NF@PAzo and NF were also prepared and used for comparison.

We investigated the ability of the NF@PAzo-Mg device to absorb, store, and release photo-thermal energy in cold environments. NF@PAzo-Mg was tailored into finger sleeves as a flexible, wearable thermal management device and placed on a model hand and human hand to realise the reversible local temperature control. The model hand (with dimensions of 30 cm × 10 cm × 8 cm) with NF@PAzo-Mg textile was placed in a refrigerator at 0-4.0 °C for further investigation.

The self-heating performance of the flexible NF@PAzo-Mg textile on the human hand was demonstrated by wearing the fabric as a self-heated glove outdoors (temperature of -5.0 to -3.0 °C, humidity of 10.0% and wind speed of 5.5–7.9 m s^{-1}).

2. Supplementary Figures

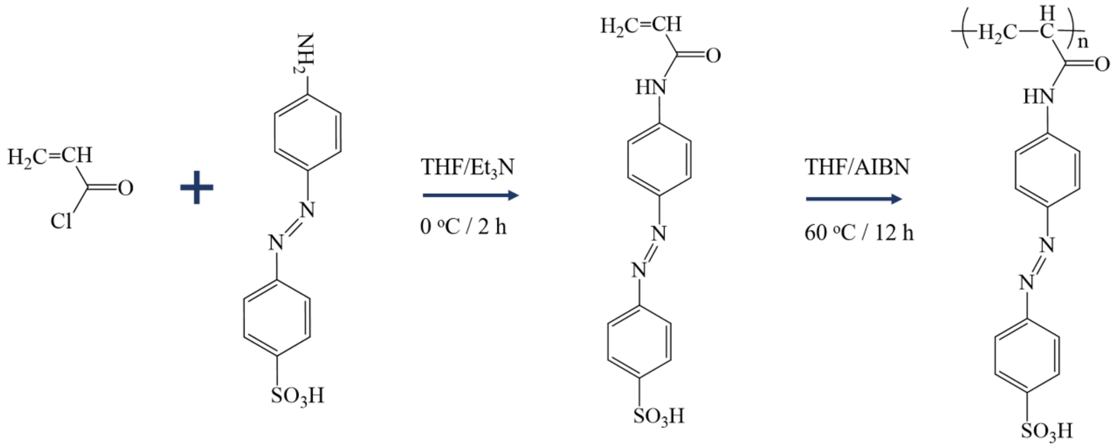

Figure S1. Chemical synthesis scheme for generating the homopolymer (PAzo) by the radical polymerization of an azobenzene monomer.

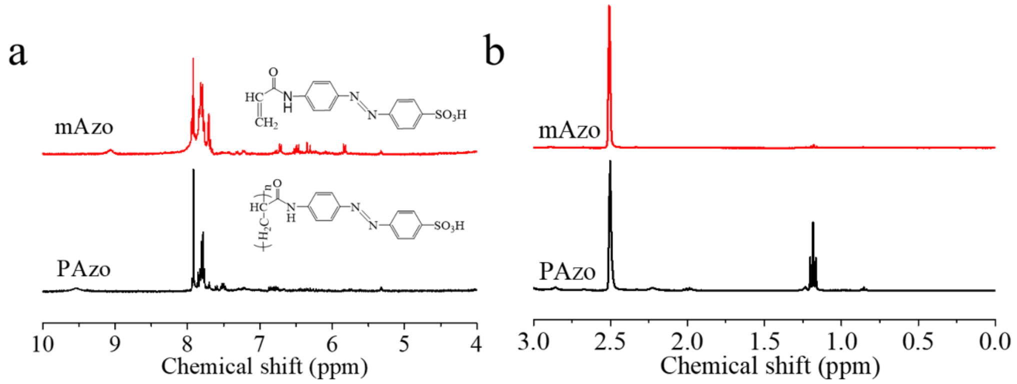

Figure S2. Partial ^{1}H NMR spectra of the mAzo and PAzo samples (samples were taken in the solid state and dissolved in dimethyl sulfoxide-d6).

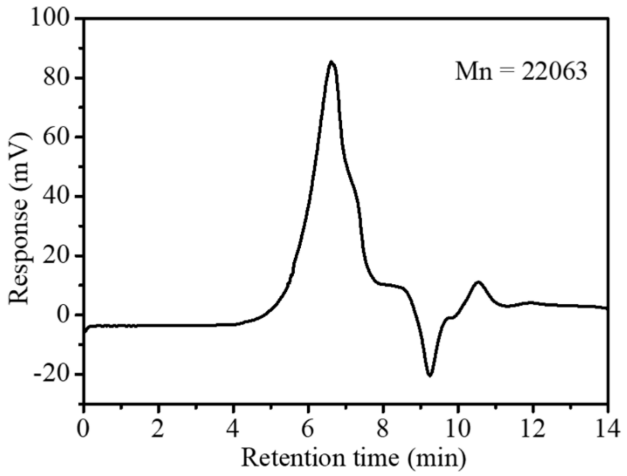

Figure S3. Gel permeation chromatography (GPC) traces for PAzo.

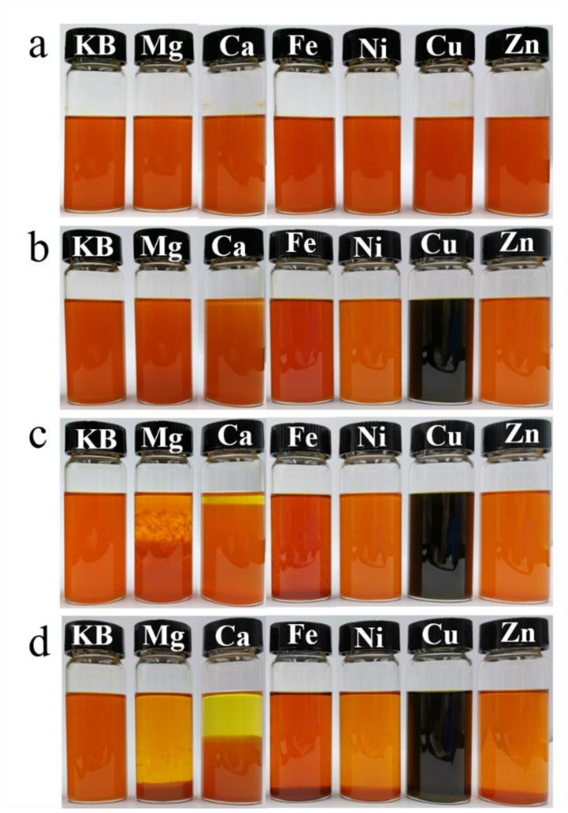

Figure S4. Time-evolved photographs of PAzo in DCM/ethanol (1:1, 20 mL) at 2.5 mg mL^{-1} in darkness after adding the same molar amount of MCl_n ($MgCl_2$, $CaCl_2$, $FeCl_3$, $NiCl_2$, $CuCl_2$, $ZnCl_2$). The MCl_n to PAzo molar ratio is 1:1, and KB is the PAzo solution without the addition of MCl_n. (a) Before addition of MCl_n. (b) 0.0 h, (c) 0.5 h, (d) 1.0 h after addition of MCl_n.

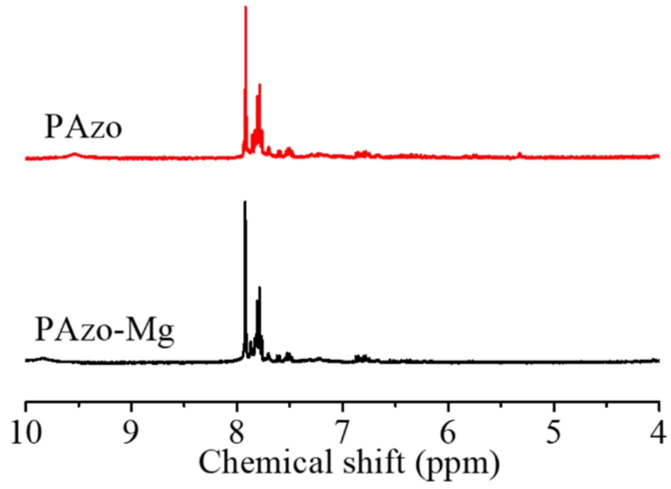

Figure S5. Partial ^1H NMR spectra of the PAzo and PAzo-Mg samples. The chemical shift changes slightly before and after adding $MgCl_2$.

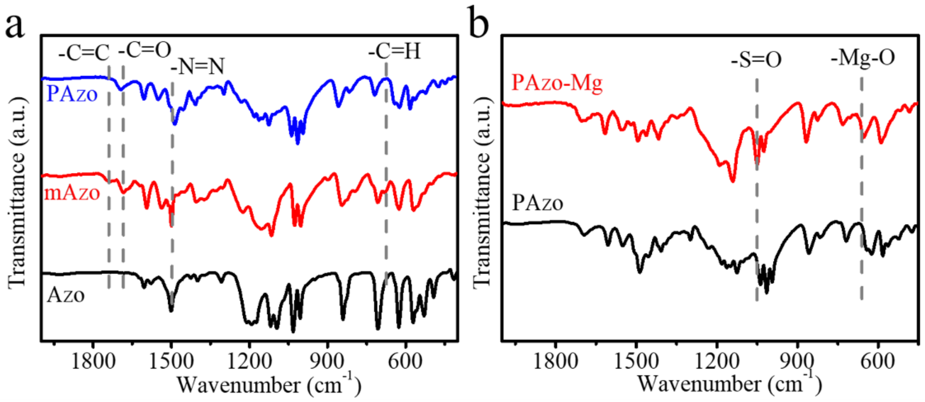

Figure S6. FT-IR spectra of Azo, mAzo, PAzo, and PAzo-Mg. The band at 650 cm^{-1} is attributed to -Mg-O stretching, that at 1050 cm^{-1} corresponds to -S=O stretching of $-SO_3H$, and a peak at 1500 cm^{-1} corresponds to -N=N.

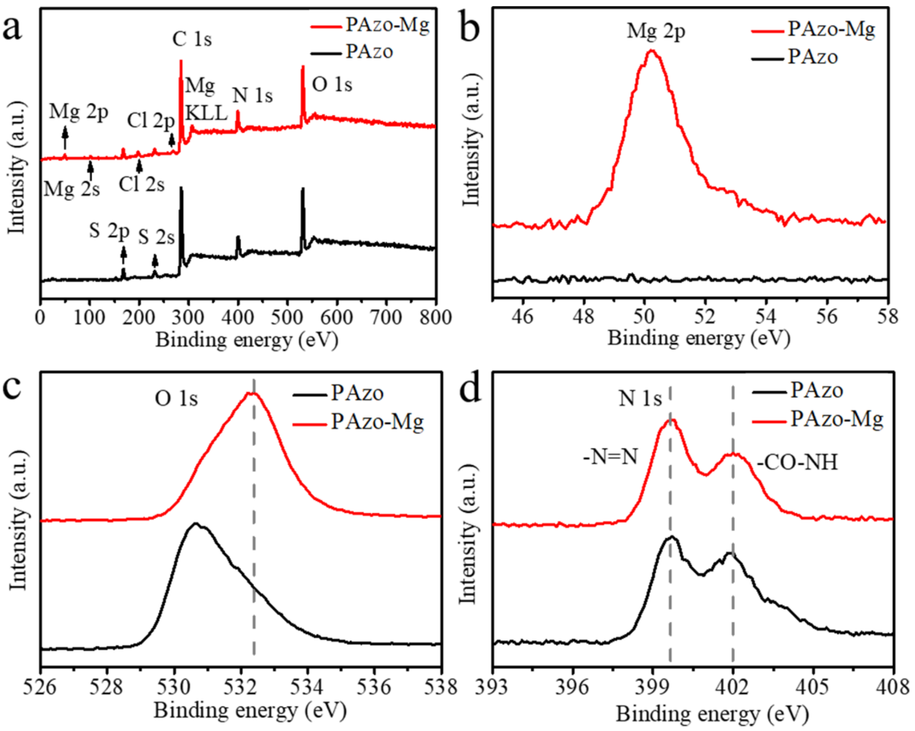

Figure S7. (a) Wide-scan survey XPS spectra of PAzo and PAzo-Mg. (b) Mg 2p region spectra, (c) O 1s region spectra, (d) N 1s region spectra.

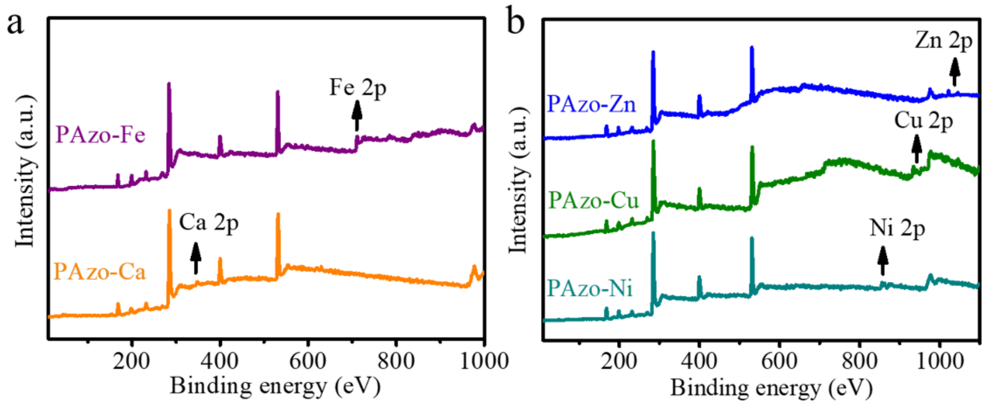

Figure S8. Wide-scan survey XPS spectra of (a) PAzo-Ca and PAzo-Fe, (b) PAzo-Ni, PAzo-Cu, and PAzo-Zn.

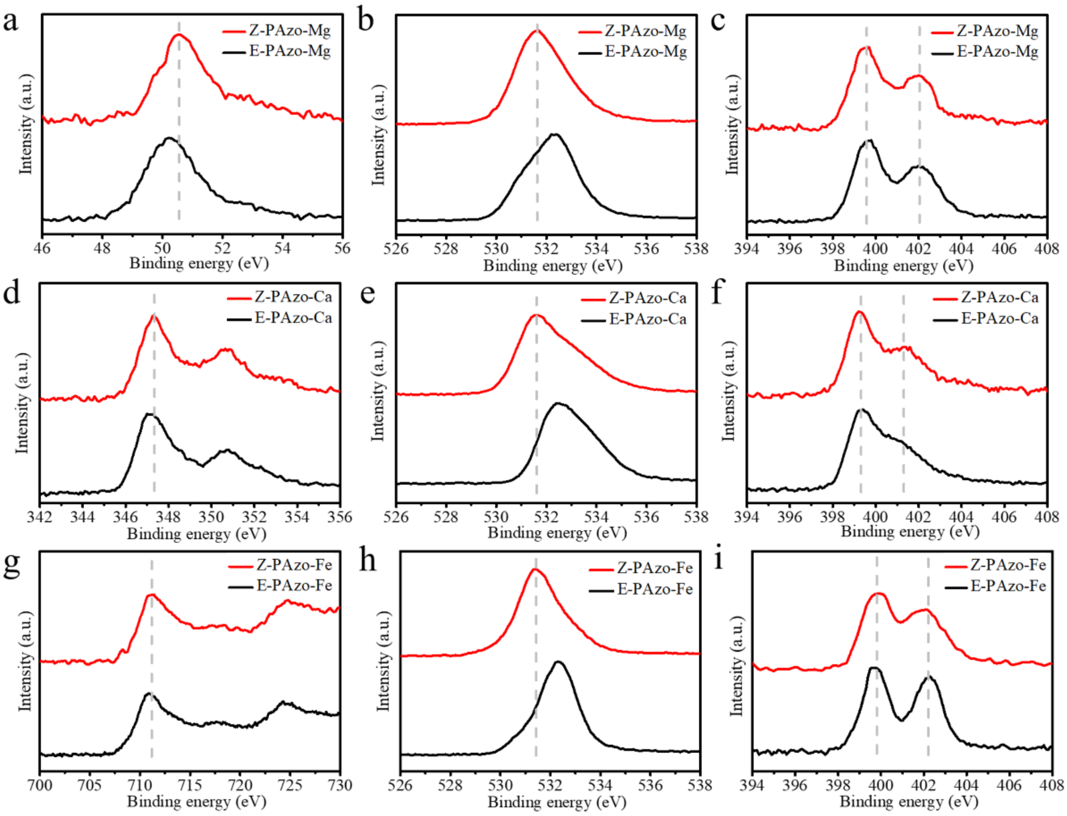

Figure S9. (a) Mg 2p, (b) O 1s, (c) N 1s region spectra of the E- and Z-PAzo-Mg. (d) Ca 2p, (e) O 1s, (f) N 1s region spectra of E- and Z-PAzo-Ca. (g) Fe 2p, (h) O 1s, (i) N 1s region spectra of E- and Z-PAzo-Fe.

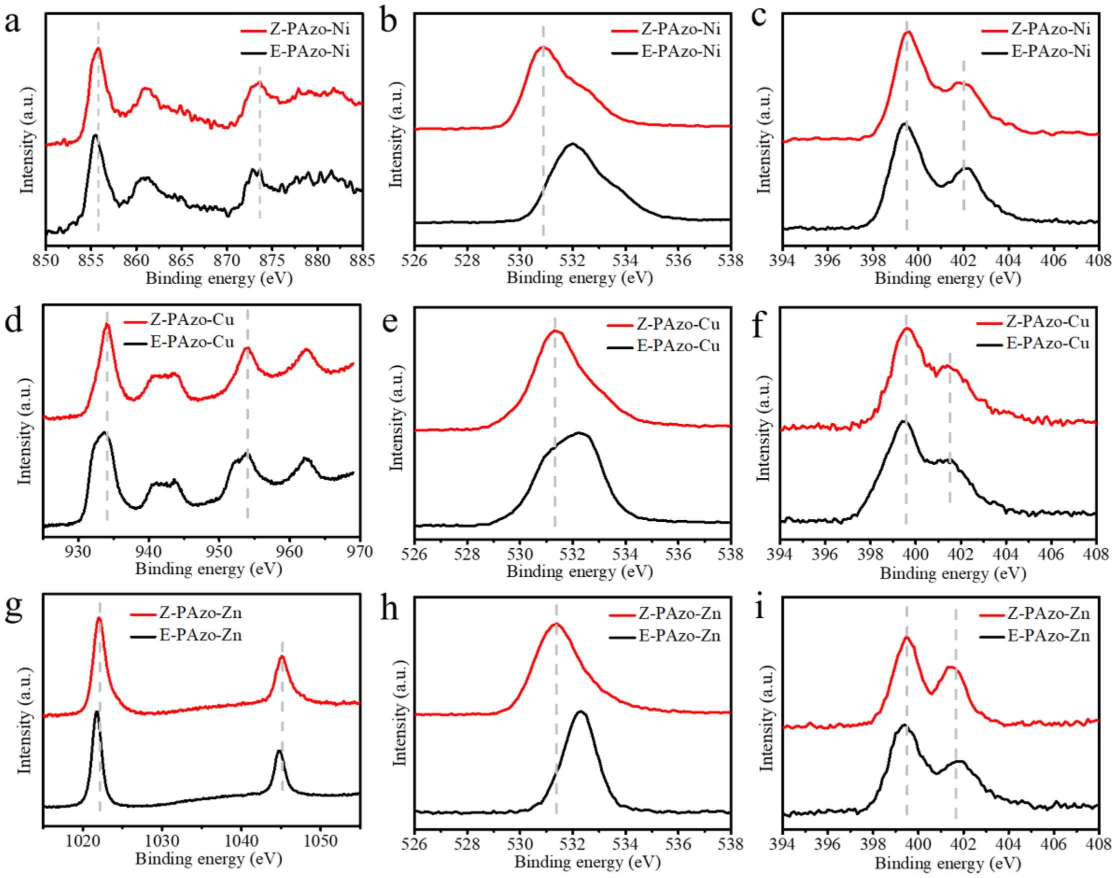

Figure S10. (a) Ni 2p, (b) O 1s, (c) N 1s region spectra of E- and Z-PAzo-Ni. (d) Cu 2p, (e) O 1s, (f) N 1s region spectra of E- and Z-PAzo-Cu. (g) Zn 2p, (h) O 1s, (i) N 1s region spectra of E- and Z-PAzo-Zn.

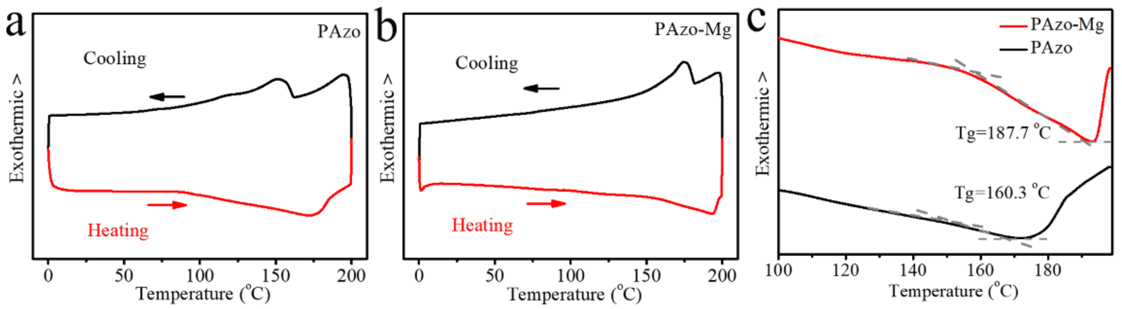

Figure S11. Second heating and second cooling DSC curves of (a) PAzo and (b) PAzo-Mg. (c) Glass transition temperature (Tg) of PAzo and PAzo-Mg obtained from (a, b).

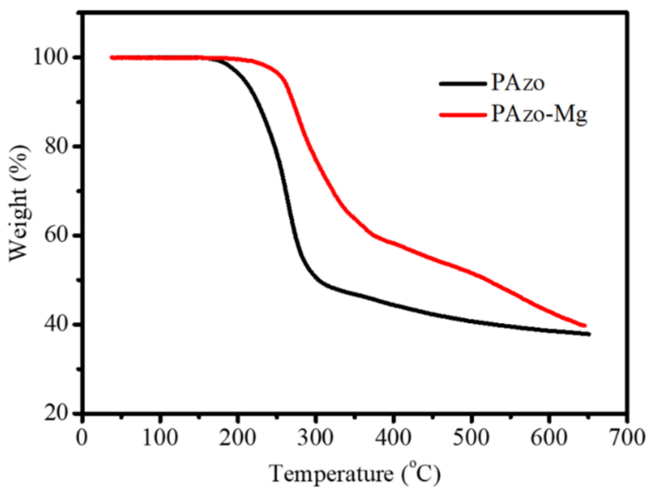

Figure S12. Thermogravimetric analysis (TGA) of PAzo and PAzo-Mg.

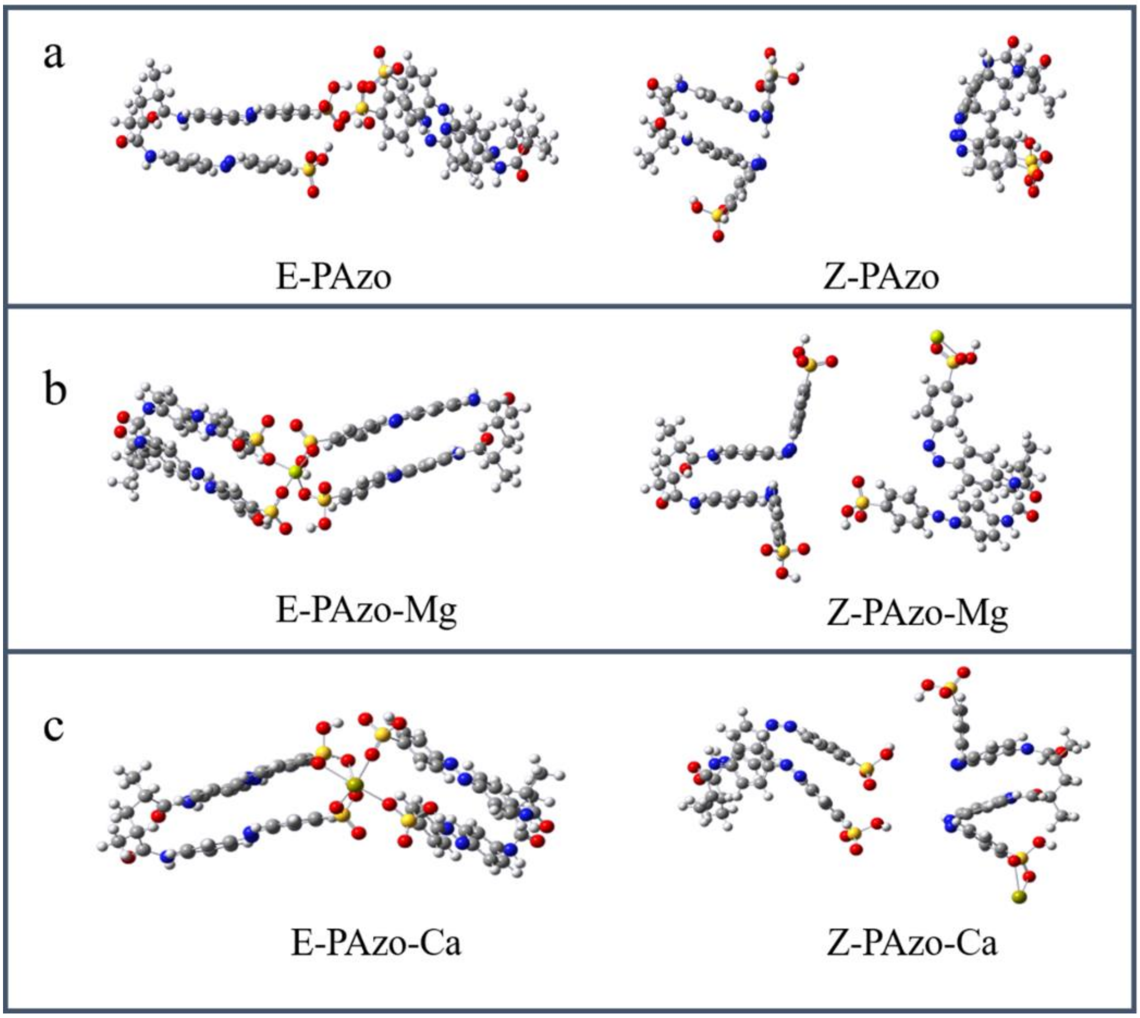

Figure S13. Optimized geometric structure of the (a) E- and Z-PAzo, (b) E- and Z-PAzo-Mg, (c) E- and Z-PAzo-Ca.

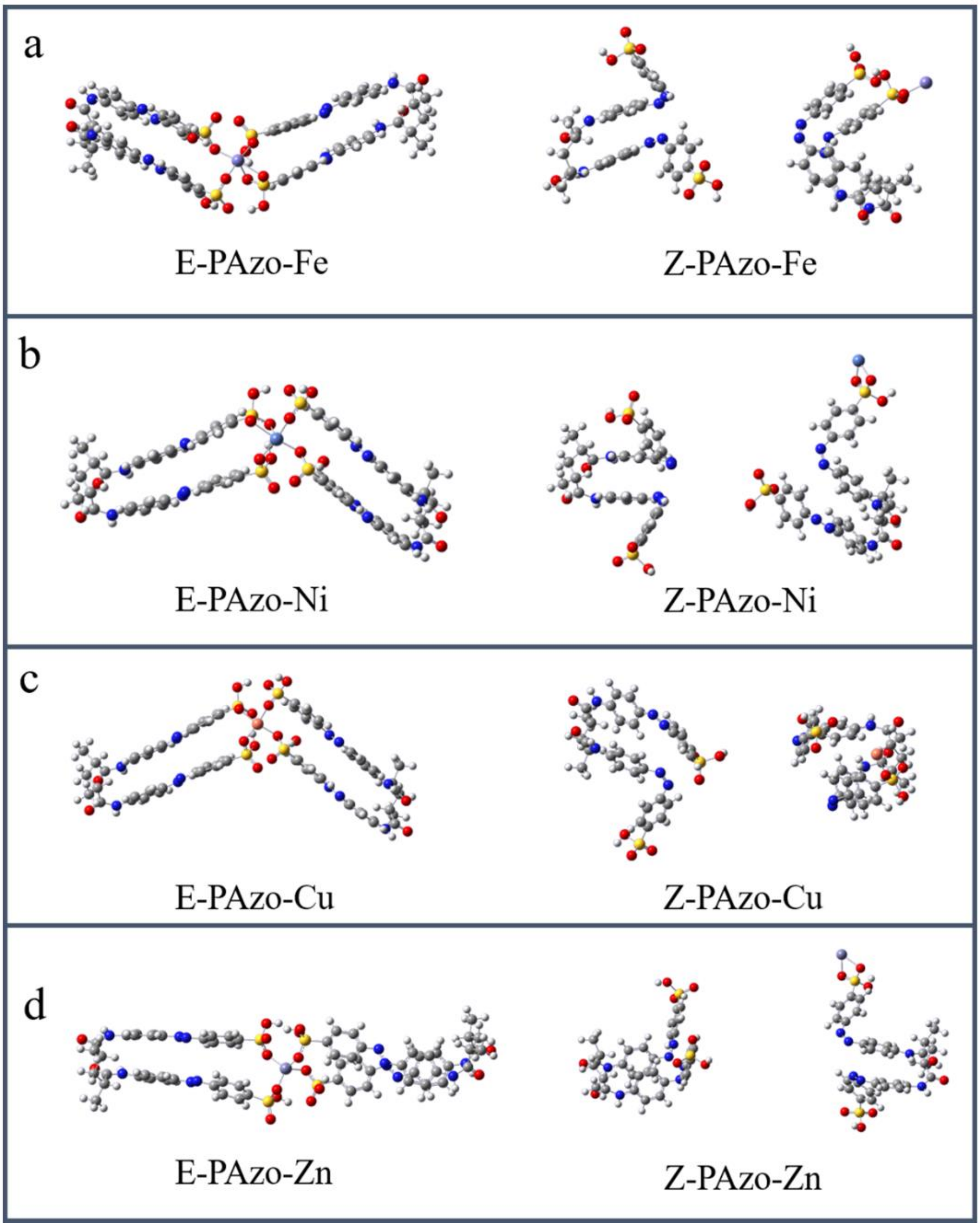

Figure S14. Optimized geometric structure of the (a) E- and Z-PAzo-Fe, (b) E- and Z-PAzo-Ni, (c) E- and Z-PAzo-Cu, (d) E- and Z-PAzo-Zn.

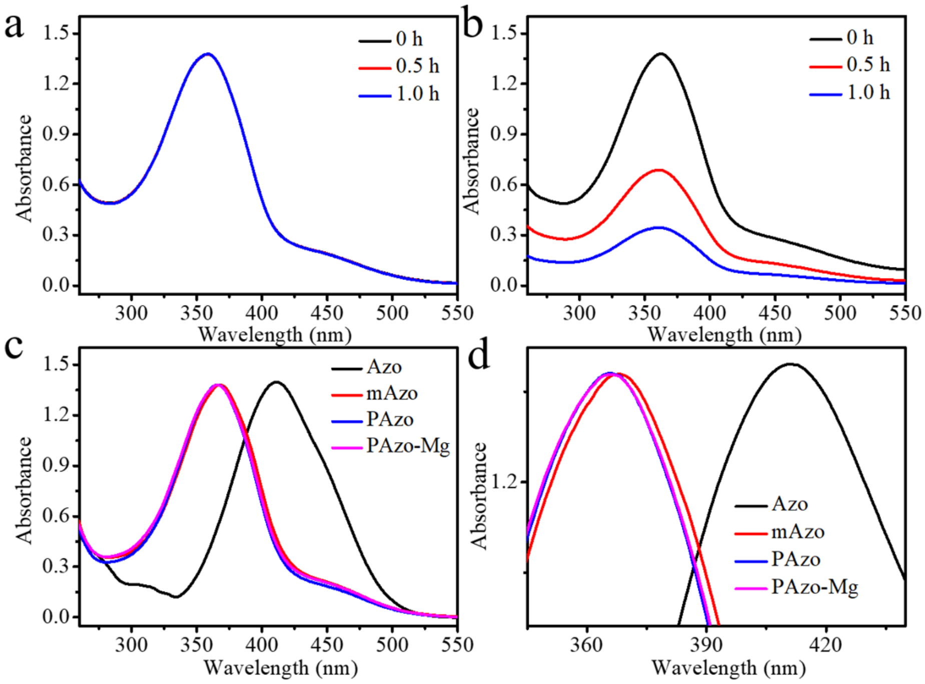

Figure S15. (a) Time-evolved UV-Vis absorption spectra of PAzo in DCM/ethanol (1:1, 20 mL) at 0.05 mg mL^{-1}. (b) Time-evolved UV-Vis absorption spectra of PAzo after adding $MgCl_2$. (c, d) UV-Vis absorption spectra of Azo, mAzo, PAzo and PAzo-Mg dissolved in DMSO at 0.05 mg mL^{-1}.

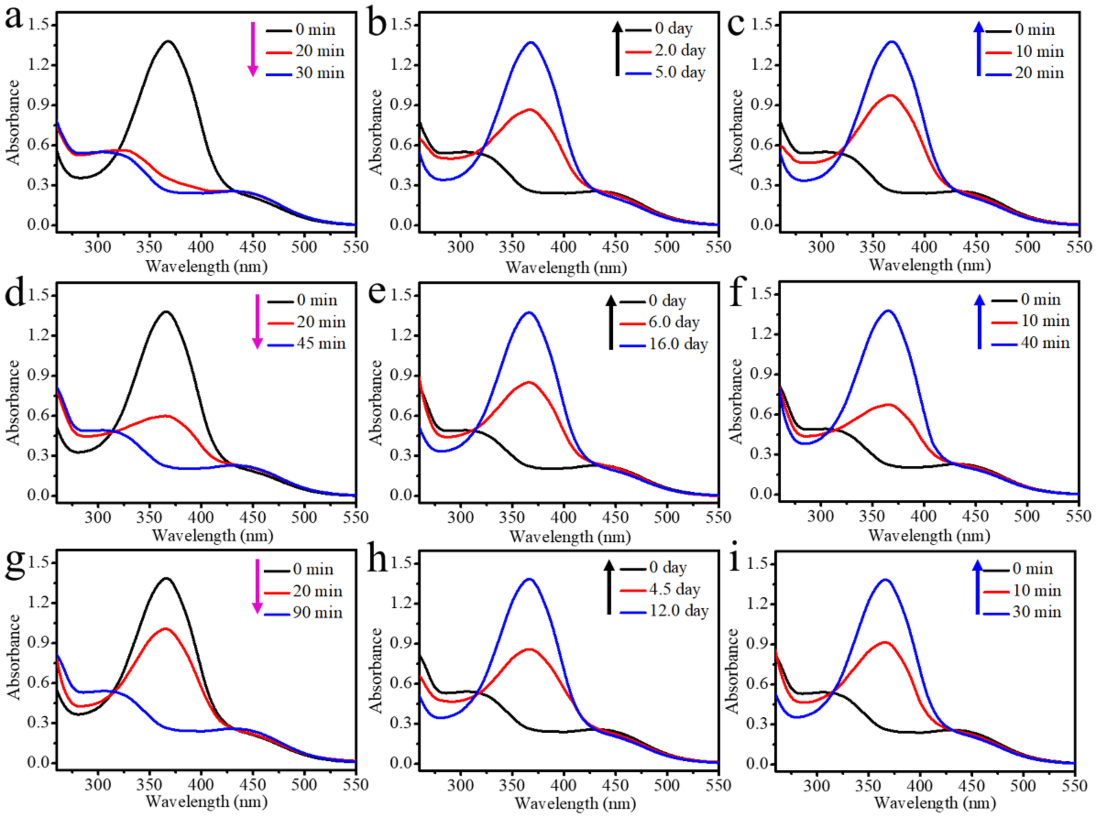

Figure S16. Time-evolved UV−Vis absorption spectra of mAzo, PAzo, and PAzo-Mg with (a, d, g) 365 nm UV irradiation, (b, e, h) in darkness and (c, f, i) 450 nm blue-light irradiation. The samples were taken in the solid state and dissolved in DMSO, and the spectra were normalized with respect to the isosbestic point at 312 nm and 432 nm. (a, b, c) mAzo, (d, e, f) PAzo, and (g, h, i) PAzo-Mg.

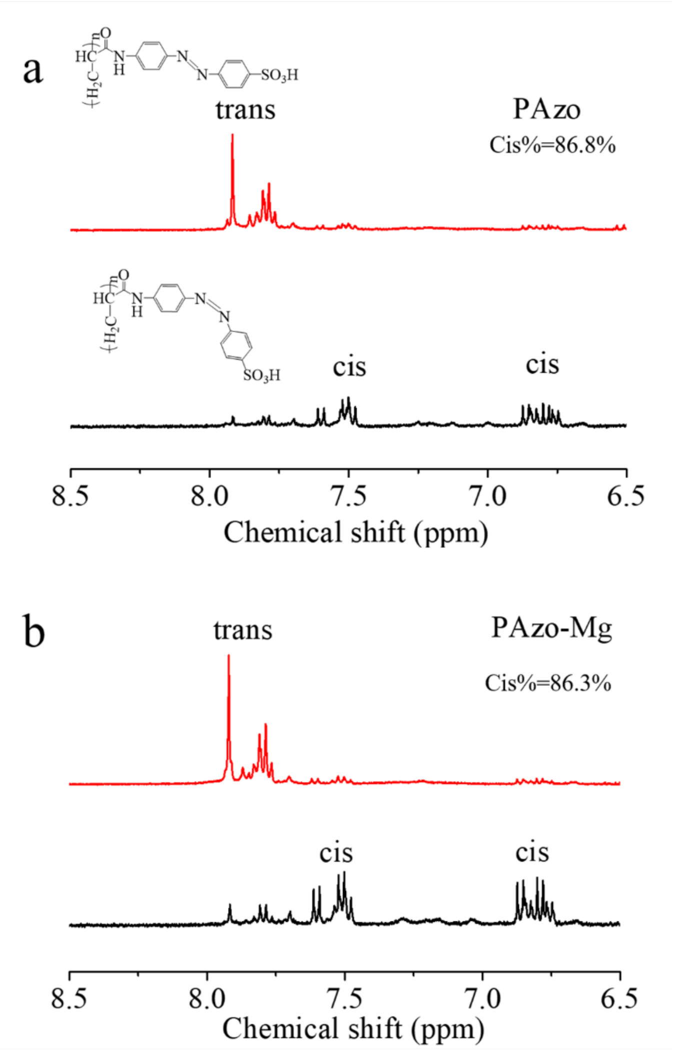

Figure S17. Partial ^{1}H NMR spectra of the trans/cis PAzo and PAzo-Mg samples. The content of cis isomer in PAzo is 86.8% after UV irradiation. The content of cis isomer in PAzo-Mg is 86.3% after UV irradiation.

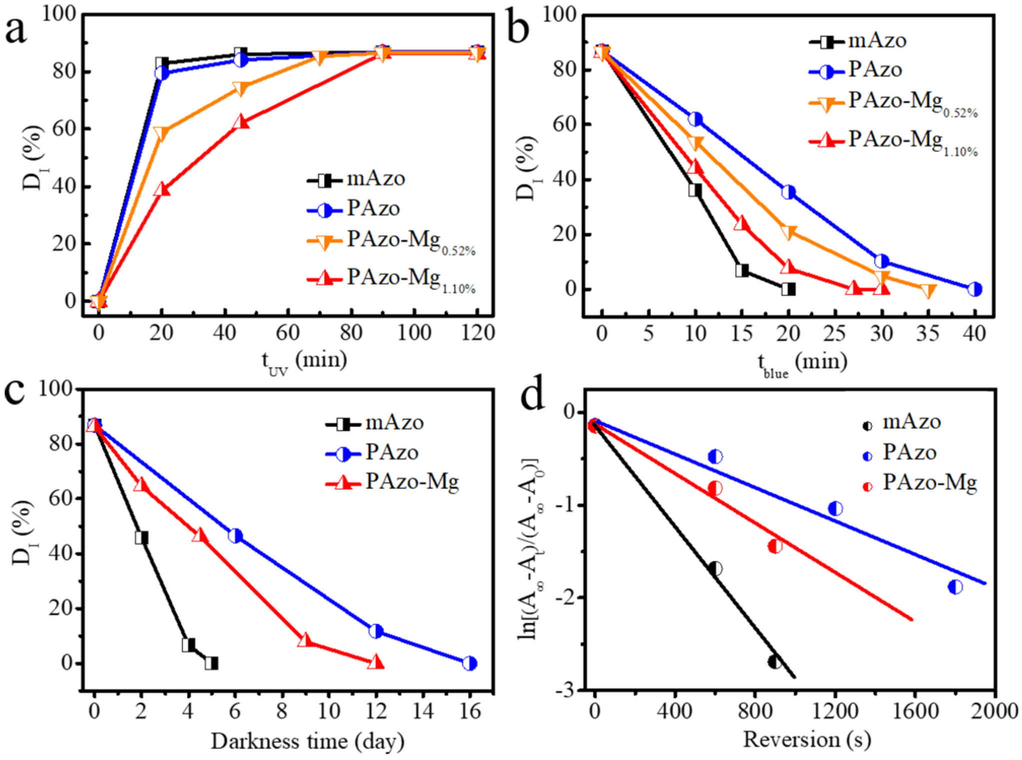

Figure S18. Isomerization degrees (D_I) of mAzo, PAzo, PAzo-Mg$_{0.52\%}$, and PAzo-Mg-$_{1.10\%}$ under (a) UV and (b) blue-light irradiation for different times (t_{UV} and t_{blue}). (c) D_I of mAzo, PAzo, and PAzo-Mg in darkness for different times. (d) First-order rate constants for Z-to-E reversion (k_{rev}) under blue-light irradiation for mAzo, PAzo, and PAzo-Mg. Note: here PAzo-Mg is the PAzo-Mg$_{1.10\%}$ in (a, b).

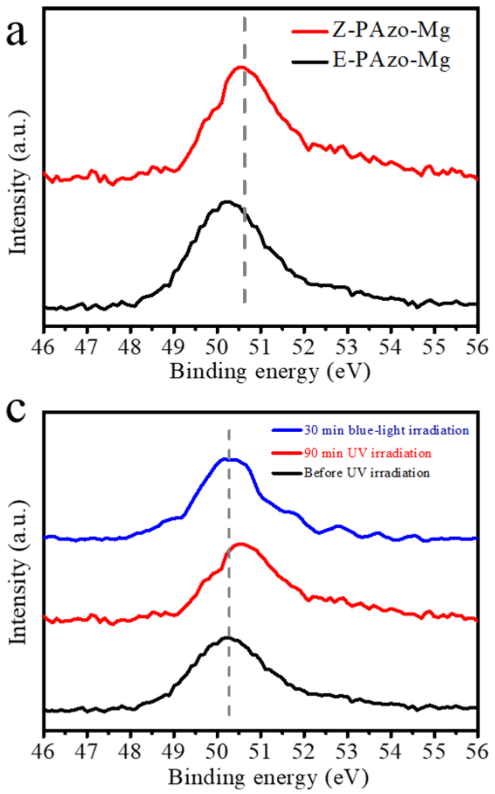

Figure S19. (a) Mg 2p, (b) O 1s region spectra of E-PAzo, E- and Z-PAzo-Mg. (c) Mg 2p, (d) O 1s region spectra of PAzo-Mg before UV irradiation, 90 min UV irradiation, 30 min blue-light irradiation.

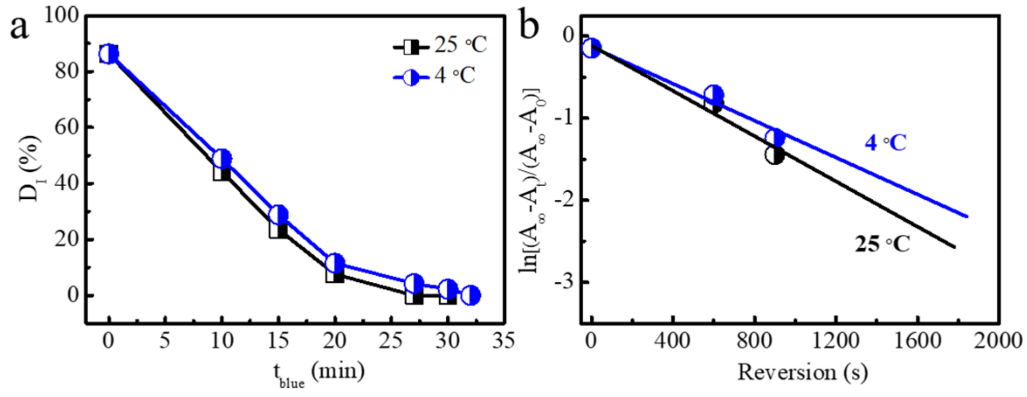

Figure S20. (a) Isomerization degrees (D_I) of PAzo-Mg under blue-light irradiation for diff erent times at 4.0 °C and 25.0 °C. (b) First-order rate constants for Z-to-E reversion (krev) under blue light irradiation for PAzo-Mg at 4.0 °C and 25.0 °C.

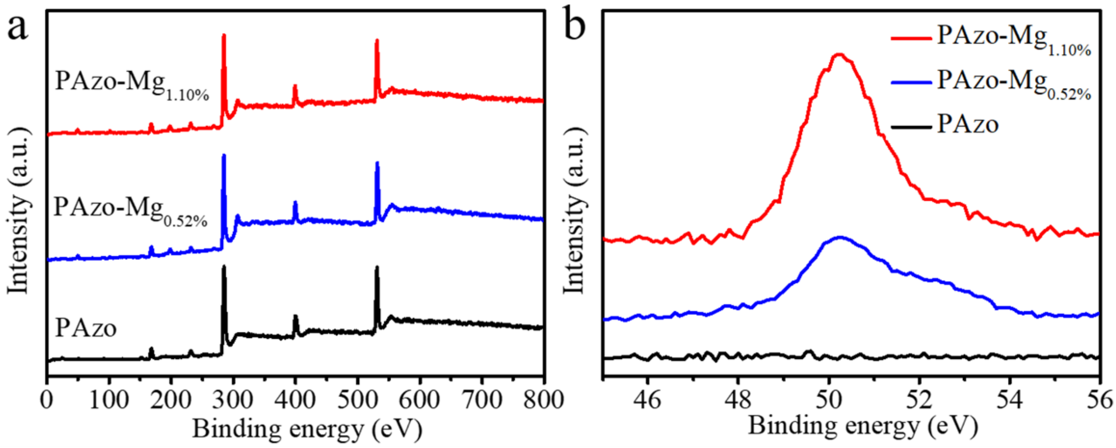

Figure S21. (a) Wide-scan survey XPS spectra and (b) Mg 2p region spectra of PAzo, PAzo-$Mg_{0.52\%}$, and PAzo-$Mg_{1.10\%}$. The contents of the Mg element in PAzo-Mg are 0.0%, 0.52%, and 1.10%.

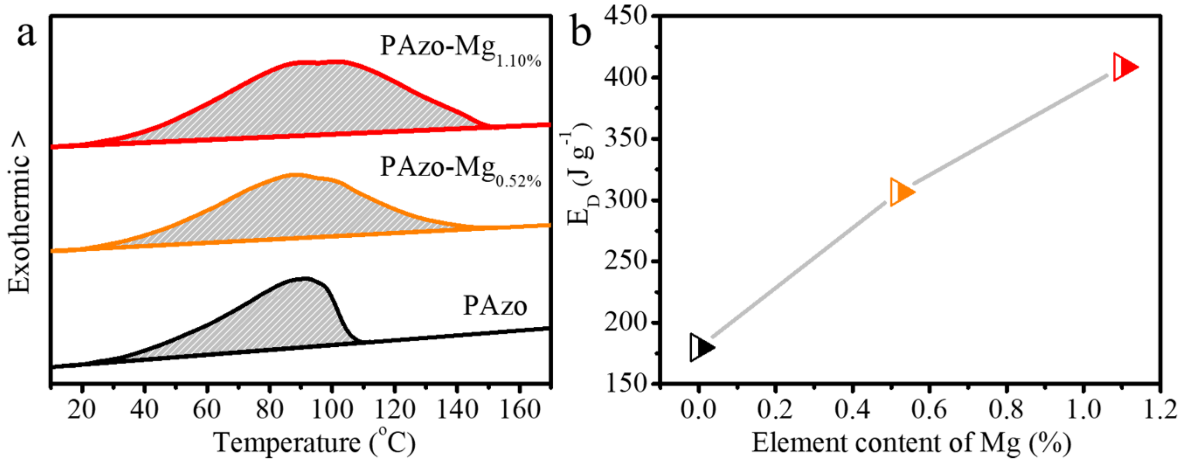

Figure S22. (a) First heating DSC results of PAzo, PAzo-$Mg_{0.52\%}$, and PAzo-$Mg_{1.10\%}$ after UV charging for 90 min. (b) Corresponding E_D with the element content of Mg.

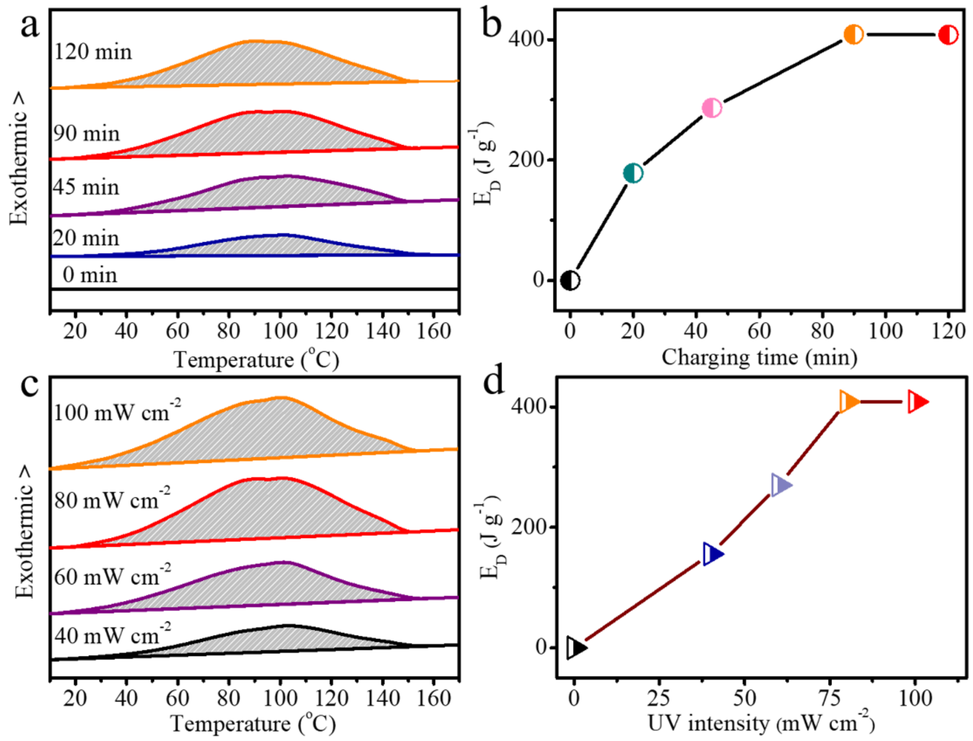

Figure S23. (a) First heating DSC results of PAzo-Mg solid charged in darkness for different times at an intensity of 80 mW cm^{-2} of UV light. (b) Relationship between E_D and UV charging time. (c) First heating DSC results of PAzo-Mg solid charged in darkness at different UV light intensities for 90 min. (d) Relationship between the E_D and UV light intensity.

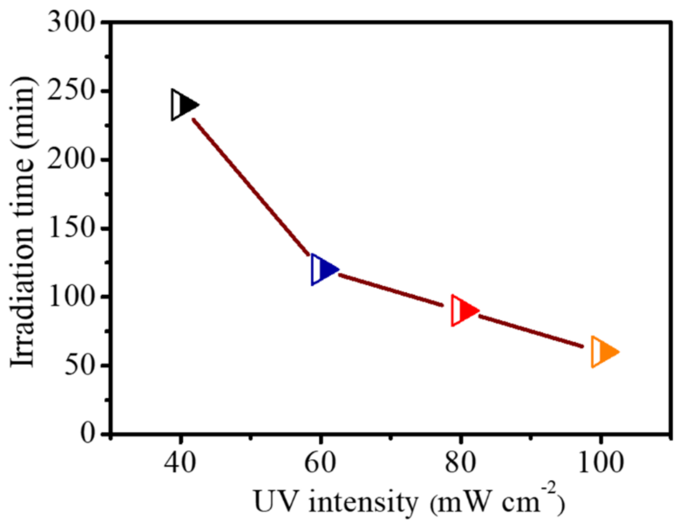

Figure S24. Relationship between the UV light intensity and irradiation time during the charging process when the photostationary state is reached.

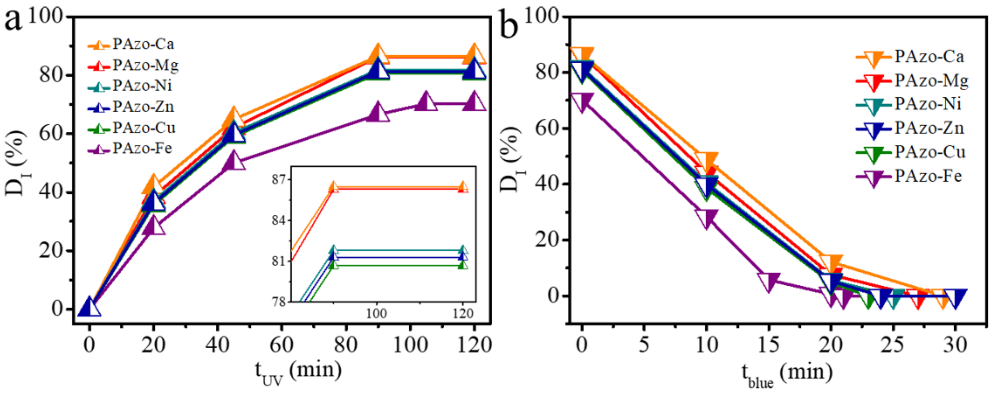

Figure S25. D_I of PAzo-M (M = Mg, Ca, Ni, Zn, Cu, and Fe) at different times when irradiated (a) by UV and (b) by blue-light at room temperature (25.0 °C).

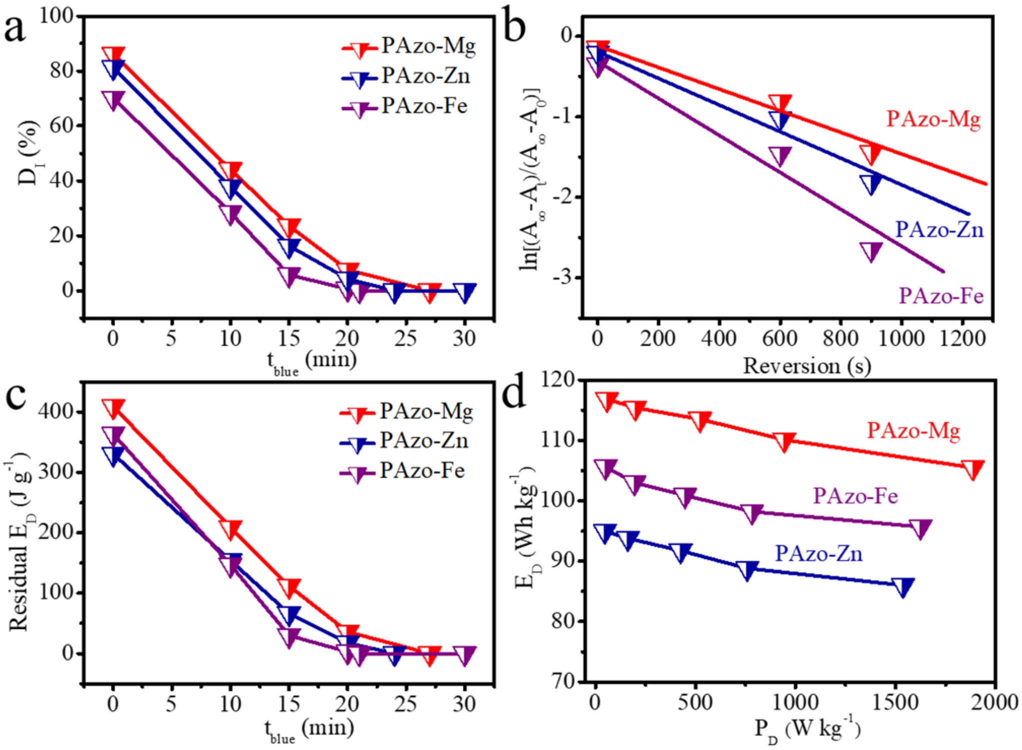

Figure S26. (a) D_I, (b) first-order rate constants for Z-to-E reversion (krev), and (c) residual E_D of PAzo-Mg, PAzo-Zn, PAzo-Fe under blue-light irradiation for different times at 25.0 °C. (d) Relationship between the E_D and power density (P_D) of PAzo-Mg, PAzo-Zn, and PAzo-Fe.

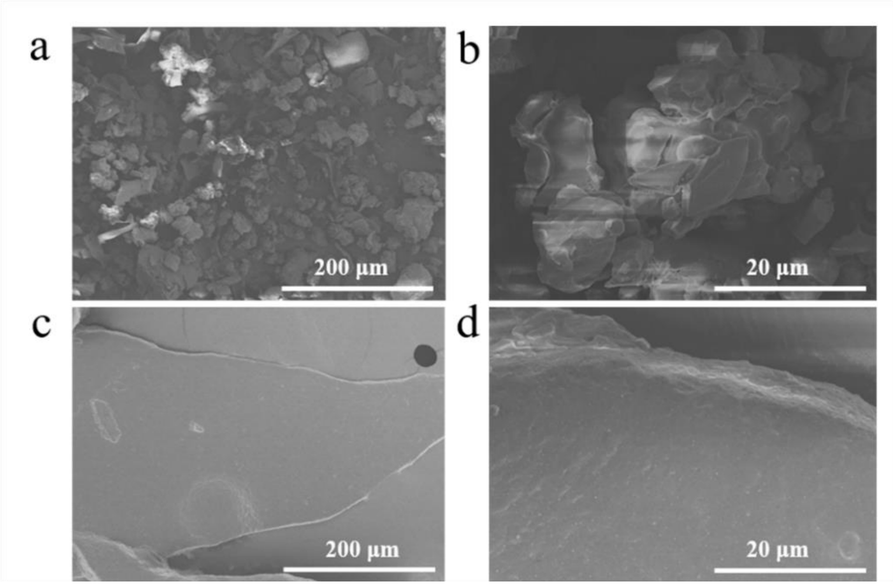

Figure S27. SEM of (a, b) PAzo and (c, d) PAzo-Mg.

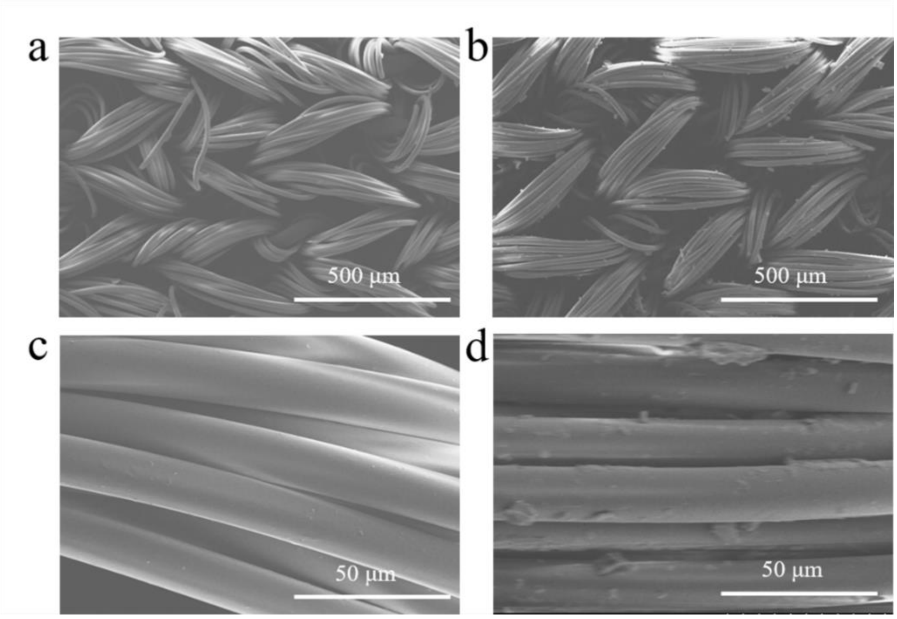

Figure S28. SEM of (a, c) the commercial nylon fabric and (b, d) NF@PAzo-Mg.

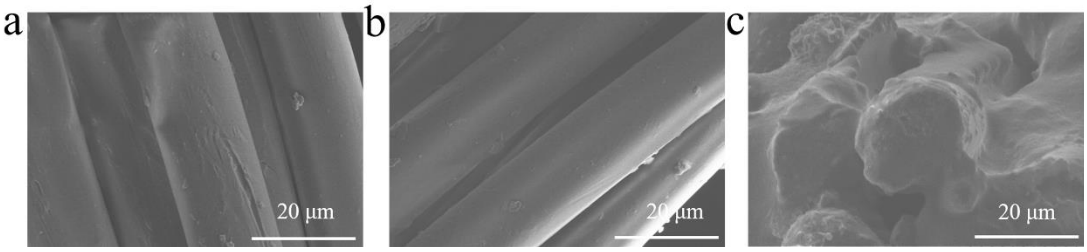

Figure S29. SEM of NF@PAzo-Mg immersed in (a) 1 mg mL^{-1}, (b) 10 mg mL^{-1}, and (c) 20 mg mL^{-1} PAzo-Mg solutions.

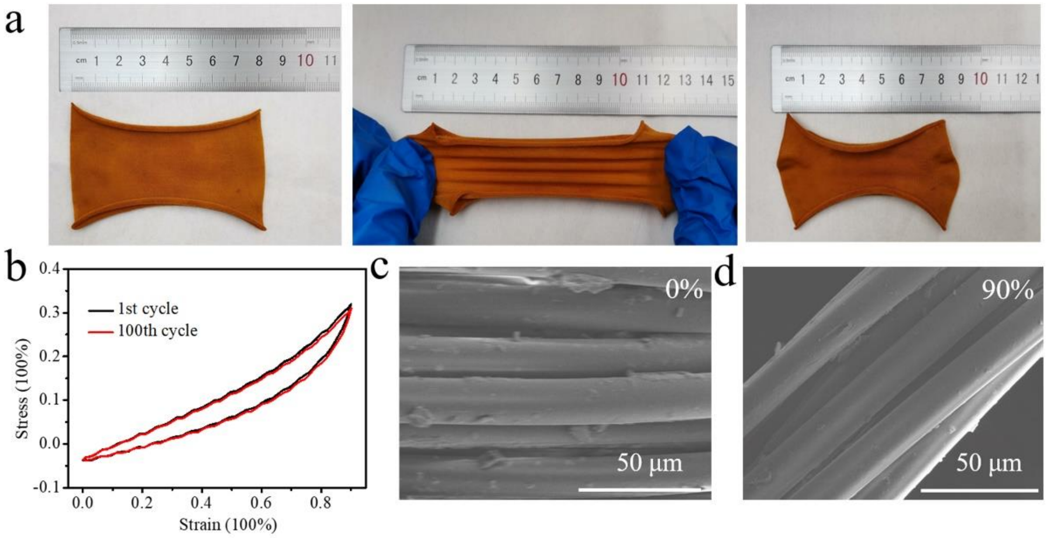

Figure S30. (a) Photographs of the stretched NF@PAzo-Mg product. It can be restored when stretched to approximately twice its length. (b) Cycle stress-strain of NF@PAzo-Mg with stretching before and after 100 cycles. SEM of NF@PAzo-Mg with (c) 0% and (d) 90% strain after stretching.

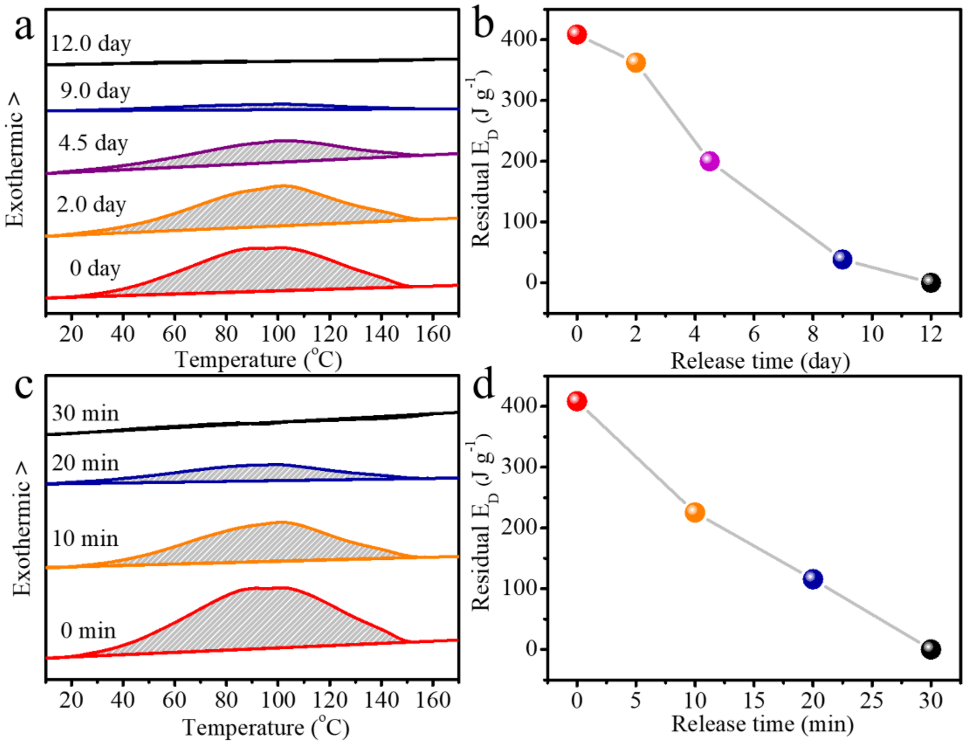

Figure S31. First heating DSC results of PAzo-Mg discharged (a) in darkness and (c) under blue-light irradiation for different times. The residual E_D of PAzo-Mg (b) in darkness and (d) under blue-light irradiation for different times.

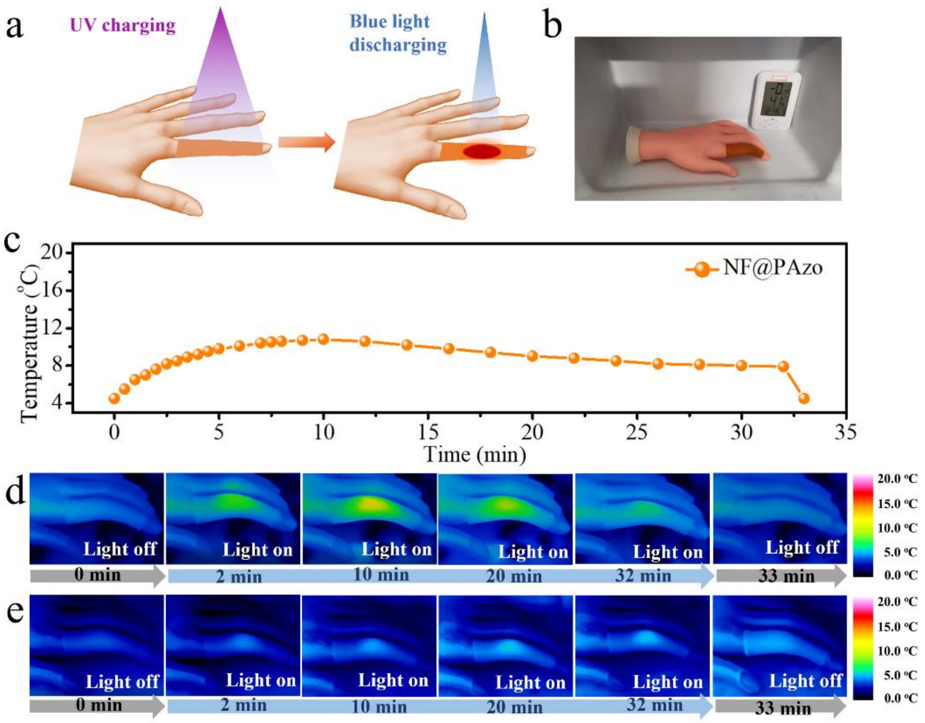

Figure S32. (a) Schematic of the NF@PAzo-Mg wearable thermal management device under UV charging and blue-light discharging. (b) Photograph of NF@PAzo-Mg wearable thermal management device at 0.0–4.0 °C. (c) Time-evolved temperature at the center of the NF@PAzo under 40 mW cm^{-2} blue-light irradiation (light on) for the heat release at 0.0–4.0 °C for 32 min, and in darkness (light off) for the next 1 minute. Time-evolved IR thermal images of (d) NF@PAzo and (e) the nylon fabric under alternate 40 mW cm^{-2} blue-light irradiation (light on) and in darkness (light off) at 0.0–4.0 °C.

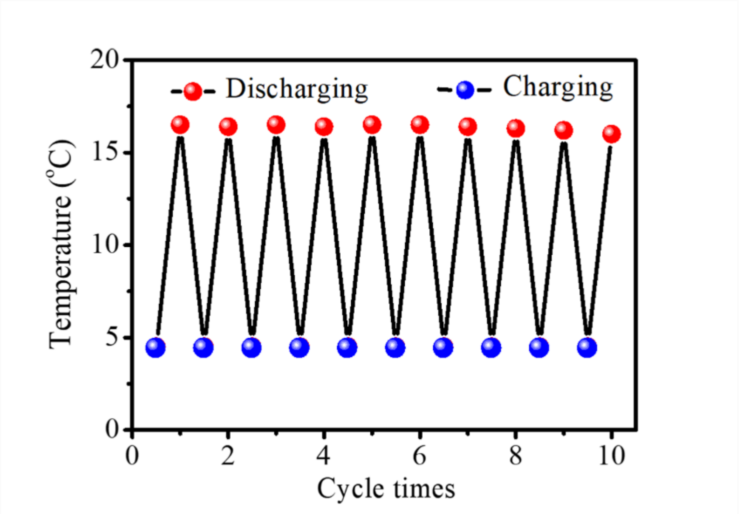

Figure S33. Maximum temperature of the NF@PAzo-Mg wearable thermal management device under 10 cycles of alternate UV (90 min, charging) and blue-light (30 min, discharging) irradiation at 0.0–4.0 °C.

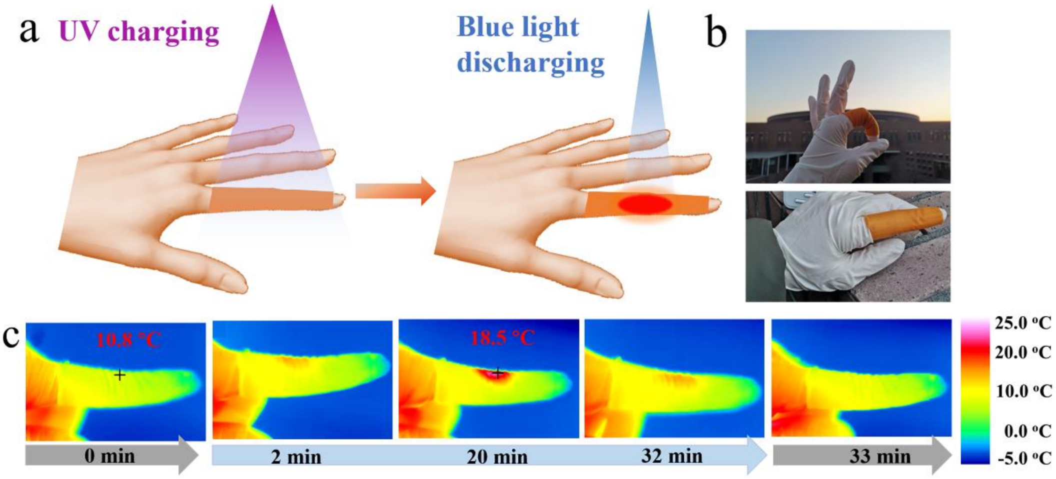

Figure S34. (a) Schematic of the NF@PAzo-Mg wearable thermal management device under UV charging and blue-light discharging. (b) Photograph of the NF@PAzo-Mg wearable thermal management device at -5.0 to -3.0 °C. (c) Time-evolved IR thermal images of the NF@PAzo-Mg under alternate 40 mW cm^{-2} blue-light irradiation (light on) and in darkness (light off) at -5.0 to -3.0 °C.

3. Supplementary Tables

Table S1. Element content of PAzo and PAzo-M (M = Mg, Ca, Ni, Zn, Cu, and Fe) according to the XPS spectra.

Sample	Element content (100%)					
	C	N	O	S	Metal	Cl
PAzo	64.40	18.28	13.21	4.11	0	0
PAzo-Mg	63.65	12.36	16.56	4.18	1.10	2.15
PAzo-Ca	64.66	12.24	16.30	4.05	0.89	1.86
PAzo-Ni	63.87	12.63	16.55	3.96	0.93	2.06
PAzo-Zn	65.30	11.64	15.66	4.13	1.06	2.21
PAzo-Cu	64.88	11.89	15.88	4.21	1.07	2.07
PAzo-Fe	63.21	12.02	16.18	4.19	1.07	3.33

Table S2. DFT-calculated Gibbs free energy (ΔG) when different metal ions are interacted with PAzo.

Metal ion	ΔG	
	eV	kJ mol^{-1}
Mg^{2+}	-14.41218271	-1390.559449
Ca^{2+}	-10.78261894	-1040.360988
Ni^{2+}	-16.34287411	-1576.842209
Zn^{2+}	-16.84098977	-1624.902898
Cu^{2+}	-17.33945919	-1672.99772
Fe^{3+}	-36.55763486	-3527.263399

Table S3. DFT-calculated energy level gap (ΔE) between the E- and Z- isomers of PAzo and PAzo-M (M=Mg, Ca, Fe, Ni, Cu, Zn).

Sample	ΔE	
	eV	$J\ g^{-1}$
PAzo	2.890769673	210.7
PAzo-Mg	6.980780078	474.6
PAzo-Ca	7.492589633	503.8
PAzo-Ni	7.690552034	510.6
PAzo-Zn	6.164551784	407.3
PAzo-Cu	5.313021907	351.5
PAzo-Fe	7.981208577	518.1

Table S4. Element content of PAzo and PAzo-Mg with different molar ratios of $MgCl_2$ to PAzo according to the XPS spectra. Note: 0.52% and 1.10% are the Mg element contents in the PAzo-Mg samples, which indicate that the corresponding proportion of $-SO_3H$ groups that interacted with Mg^{2+} is 48.8% and 100.0% in the total $-SO_3H$ groups content, respectively.

Sample	Element content (100%)						χ
	C	N	O	S	Metal	Cl	%
PAzo-Mg	64.40	13.21	18.28	4.11	0	0	0.0
PAzo-$Mg_{0.52\%}$	63.91	12.80	17.43	4.26	0.52	1.08	48.8
PAzo-$Mg_{1.10\%}$	63.65	12.36	16.56	4.18	1.10	2.15	100.0

Note: χ is the corresponding proportion of $-SO_3H$ groups that interacted with Mg^{2+} with electrostatic or coordination in the total $-SO_3H$ groups content.

Table S5. Relative binding energy (E_B) of the hydrogen bond (H-O) and M-O bonds.

M-O	Coordination number	E_B	
		eV	kJ mol^{-1}
H-O		-0.964018571	-93.01327673
Mg-O	4,6	-15.58822203	-1504.029603
Ca-O	6,8	-11.90543934	-1148.696315
Ni-O	6	-17.62066555	-1700.129915
Zn-O	4,6	-18.01557861	-1738.233102
Cu-O	4,6	-18.55031509	-1789.827151
Fe-O	4,6	-37.65558817	-3633.199425

Table S6. Isomerization degree (D_I), energy density (E_D) of PAzo and PAzo-M (M=Mg, Ca, Fe, Ni, Cu, Zn).

Sample	D_I	E_D		
	%	J g^{-1}	Wh kg^{-1}	eV
PAzo	86.8	179.8	49.9	2.4677411
PAzo-Mg	86.3	408.6	113.5	6.010260662
PAzo-Ca	86.5	381.4	105.9	5.672384308
PAzo-Ni	81.8	394.2	109.5	5.938746955
PAzo-Zn	81.3	330.3	91.7	4.998704462
PAzo-Cu	80.7	283.0	78.6	4.278385241
PAzo-Fe	70.2	363.3	100.9	5.595688449

Table S7. Photothermal storage parameters related to the polymer-templated azobenzene.

Materials	state	Energy density Wh kg^{-1}	$\tau_{1/2}$ h	Ref.
Polyacrylate azobenzene	solid	29	55	*1*
Polymethacrylate-hexyl ester-azobenzene	liquid	35	12	*2*
Polymethacrylate azobenzene	solid	24-26	75	*3*
Cationic azobenzene derivatives	liquid-crystals	36	-	*4*
Azobenzene-functionalized diacetylenes	liquid-crystals	66	98	*5*
Polynorbornene-templated azobenzene	film	49	0.25	*6*
Photodynamic polymer-templated azobenzenes	solid	113.5	108	*Our work*

4. Supplementary Equations

Equation S1

For PAzo:

$$\Delta E = \Delta E_I + \Delta E_H$$

ΔE is the energy gap. ΔE_I is the isomerization energy between E- and Z-isomers without molecular interaction. E_H is hydrogen bond enthalpy.

Equation S2

$$\ln\left(\frac{A_\infty - A_t}{A_\infty - A_0}\right) = -\kappa_{rev} t$$

A_0 is the absorption intensity of PAzo-Mg in the metastable state (cis-rich) irradiated by UV light. A_t is the absorption intensity of PAzo-Mg reversal for “t” time. A_∞ is the absorption intensity of PAzo-Mg in the stable state (trans-rich). κ_{rev} represents the first-order rate constant.

Equation S3

The D_I of the PAzo-M is estimated as the percentage change of absorbance at the peak π- π^* following the equation: [11,12]

$$D_I = \frac{A_t - A_\infty}{A_0 - A_\infty} \times 100\%$$

A_0 is the absorption intensity of PAzo-Mg in the metastable state (cis-rich) irradiated by UV light. A_t is the absorption intensity of PAzo-Mg reversal for “t” time. A_∞ is the absorption intensity of PAzo-Mg in the stable state (trans-rich). The D_I of cis-rich state was measured to be 86.3% for PAzo-Mg and 86.8% for PAzo according to the ^1H NMR spectra and the trans-rich state was assumed to be ~100% trans.

5. References

[1] D. Zhitomirsky, E. Cho, J. C. Grossman, *Adv. Energy Mater*. **2016**, *6*, 1502006.

[2] A. K. Saydjari, P. Weis, S. Wu, *Adv. Energy Mater*. **2017**, *7*, 1601622.

[3] D. Zhitomirsky, J. C. Grossman, *ACS Appl. Mater. Interfaces*. **2016**, *8*, 26319-26325.

[4] K. Ishiba, M. Morikawa, Chie. Chikara, T. Yamada, K. Iwase, M. Kawakita, N. Kimizuka, *Angew. Chem. Int. Ed*. **2015**, *54*, 1532-1536.

[5] G. D. Han, S. S. Park, Y. Liu, D. Zhitomirsky, E. Cho, M. Dincă, J. C. Grossman, *J. Mater. Chem. A* **2016**, *4*, 16157-16165.

[6] L. Fu, J. Yang, L. Dong, H. Yu, Q. Yan, F. Zhao, F. Zhai, Y. Xu, Y. Dang, W. Hu, Y. Feng, W. Feng, *Macromolecules* **2019**, *52*, 4222-4231.
